# Rapid, comprehensive, and affordable mycobacterial diagnosis with whole-genome sequencing: a prospective study

**DOI:** 10.1016/S2213-2600(15)00466-X

**Published:** 2016-01

**Authors:** Louise J Pankhurst, Carlos del Ojo Elias, Antonina A Votintseva, Timothy M Walker, Kevin Cole, Jim Davies, Jilles M Fermont, Deborah M Gascoyne-Binzi, Thomas A Kohl, Clare Kong, Nadine Lemaitre, Stefan Niemann, John Paul, Thomas R Rogers, Emma Roycroft, E Grace Smith, Philip Supply, Patrick Tang, Mark H Wilcox, Sarah Wordsworth, David Wyllie, Li Xu, Derrick W Crook

**Affiliations:** a Microbiology and Infectious Diseases, Nuffield Department of Clinical Medicine, John Radcliffe Hospital, University of Oxford, Oxford, UK; b Health Economics Research Centre, Nuffield Department of Population Health, University of Oxford, Oxford, UK; c Department of Computer Science, University of Oxford, Oxford, UK; d Brighton and Sussex University Hospitals NHS Trust, Brighton, UK; e Public Health England Regional Centre for Mycobacteriology, Birmingham Heartlands Hospital NHS Foundation Trust, Birmingham, UK; f Leeds Teaching Hospitals NHS Trust, Leeds, UK; g Université de Lille, Centre national de la recherche scientifique Unité mixte de recherche 8204, Institut national de la santé et de la recherche médicale U1019, Centre Hospitalier Universitaire, and Center for Infection and Immunity of Lille, Institut Pasteur de Lille, Lille, France; h Genoscreen, Lille, France; i British Columbia Public Health Microbiology and Reference Laboratory, Vancouver, Canada; j Molecular Mycobacteriology, Forschungszentrum Borstel, Leibniz-Zentrum für Medizin und Biowissenschaften, Schleswig-Holstein, Germany; k German Center for Infection Research, Borstel, Germany; l Department of Clinical Microbiology Trinity College Dublin and Irish Mycobacteria Reference Laboratory, St James's Hospital, Dublin, Ireland; m Public Health England, Oxford, UK

## Abstract

**Background:**

Slow and cumbersome laboratory diagnostics for *Mycobacterium tuberculosis* complex (MTBC) risk delayed treatment and poor patient outcomes. Whole-genome sequencing (WGS) could potentially provide a rapid and comprehensive diagnostic solution. In this prospective study, we compare real-time WGS with routine MTBC diagnostic workflows.

**Methods:**

We compared sequencing mycobacteria from all newly positive liquid cultures with routine laboratory diagnostic workflows across eight laboratories in Europe and North America for diagnostic accuracy, processing times, and cost between Sept 6, 2013, and April 14, 2014. We sequenced specimens once using local Illumina MiSeq platforms and processed data centrally using a semi-automated bioinformatics pipeline. We identified species or complex using gene presence or absence, predicted drug susceptibilities from resistance-conferring mutations identified from reference-mapped MTBC genomes, and calculated genetic distance to previously sequenced UK MTBC isolates to detect outbreaks. WGS data processing and analysis was done by staff masked to routine reference laboratory and clinical results. We also did a microcosting analysis to assess the financial viability of WGS-based diagnostics.

**Findings:**

Compared with routine results, WGS predicted species with 93% (95% CI 90–96; 322 of 345 specimens; 356 mycobacteria specimens submitted) accuracy and drug susceptibility also with 93% (91–95; 628 of 672 specimens; 168 MTBC specimens identified) accuracy, with one sequencing attempt. WGS linked 15 (16% [95% CI 10–26]) of 91 UK patients to an outbreak. WGS diagnosed a case of multidrug-resistant tuberculosis before routine diagnosis was completed and discovered a new multidrug-resistant tuberculosis cluster. Full WGS diagnostics could be generated in a median of 9 days (IQR 6–10), a median of 21 days (IQR 14–32) faster than final reference laboratory reports were produced (median of 31 days [IQR 21–44]), at a cost of £481 per culture-positive specimen, whereas routine diagnosis costs £518, equating to a WGS-based diagnosis cost that is 7% cheaper annually than are present diagnostic workflows.

**Interpretation:**

We have shown that WGS has a scalable, rapid turnaround, and is a financially feasible method for full MTBC diagnostics. Continued improvements to mycobacterial processing, bioinformatics, and analysis will improve the accuracy, speed, and scope of WGS-based diagnosis.

**Funding:**

National Institute for Health Research, Department of Health, Wellcome Trust, British Colombia Centre for Disease Control Foundation for Population and Public Health, Department of Clinical Microbiology, Trinity College Dublin.

## Introduction

In 2013, WHO estimated that *Mycobacterium tuberculosis* complex (MTBC) caused 9 million new active infections and 1·5 million deaths worldwide.^1^ Non-tuberculous mycobacteria also cause considerable morbidity and mortality.^2^ Protracted MTBC diagnosis and phenotypic drug susceptibility testing (DST) due to slow growth in culture contribute to reported treatment initiation delays of 8–80 days from first contact with health services, risking poor clinical outcomes and transmission control.^3^, ^4^, ^5^, ^6^ Although genotypic assays such as the Cepheid Xpert MTB/RIF (Cepheid, Sunnyvale, CA, USA) and Hain line-probe (Hain Lifescience, Nehren, Germany) assays can rapidly (less than a day) identify mycobacterial species and mutations conferring MTBC drug resistance independent of culture, they do not detect all resistance-conferring mutations and are typically still used after microbial culture.^5^, ^6^, ^7^, ^8^, ^9^ Besides identifying species and doing DST, high-income countries also genotype MTBC using mycobacterial interspersed repetitive unit-variable-number tandem repeat (MIRU-VNTR) for outbreak detection.

Findings from retrospective studies^6^, ^8^, ^9^, ^10^, ^11^, ^12^, ^13^, ^14^, ^15^, ^16^, ^17^, ^18^, ^19^ show the potential for whole-genome sequencing (WGS) to predict drug susceptibility and simultaneously track outbreaks with high resolution. WGS could replace the entire MTBC diagnostic workflow from Mycobacteria Growth Indicator Tubes (MGITs; BACTEC MGIT; Beckton Dickinson, Franklin Lakes, NJ, USA) thanks to demonstrable benefits for outbreak detection, growing knowledge bases for drug resistance-conferring mutations, and reliable DNA isolation from newly positive culture.^6^, ^8^, ^9^, ^10^, ^11^, ^12^, ^13^, ^14^, ^15^, ^16^, ^17^, ^18^, ^19^, ^20^ So far, to our knowledge, no investigators have assessed this process prospectively. In this prospective study, we compare real-time WGS with routine MTBC diagnostic workflows at Illumina MiSeq (Illumina, San Diego, CA, USA)-equipped laboratories across Europe and North America. We also do a microcosting analysis to assess the financial viability of WGS-based diagnostics.

**Research in context**

**Evidence before this study**

We searched PubMed for studies published before July 1, 2015, with no language restrictions, using the search terms “whole genome sequencing”, “diagnosis”, “infection”, “mycobacterium”, and “tuberculosis”. Full diagnosis of *Mycobacterium tuberculosis* consists of identification of the organism, establishment of antibiotic sensitivity profiles, and outbreak investigation. During the last 5 years, whole-genome sequencing (WGS) has been increasingly used to assist aspects of this diagnostic pathway. Its primary use has been outbreak investigations, for which WGS provides higher-resolution outbreak tracing than does traditional typing. WGS has begun to be used to elucidate drug resistance profiles for tuberculosis and discover new resistance-conferring mutations. In view of the slow (1–2 months) diagnostic time for tuberculosis with culture-based methods, introduction of molecular assays, including WGS, to replace aspects of the tuberculosis diagnostic pathway has been recognised to improve outbreak control and potentially expedite patient treatment. However, many barriers to widespread adoption of WGS have been raised. These barriers are high diagnostic costs and personnel efforts and an absence of automated sequence analysis pipelines and supporting IT infrastructure.

**Added value of this study**

In this study, we implement an end-to-end WGS-based diagnostic system for tuberculosis in eight laboratories in Europe and North America. Using a decentralised sequencing, centralised analysis model and a semi-automated bioinformatics pipeline, we sequenced newly positive mycobacterium cultures and generated diagnostic reports identifying the mycobacteria present, and for tuberculosis, predicted resistance to first-line and second-line antibiotics and did outbreak analysis. Prospective assessment of the WGS-based diagnostic system allows diagnostic accuracy to be directly compared with routine clinical diagnosis, which shows how WGS is scalable and how it could provide full diagnostic information weeks faster than routine clinical diagnostics could. Through a microcosting analysis comparing routine with WGS-based diagnostics, the financial feasibility of WGS-based diagnostics in high-income countries is established.

**Implications of all the available evidence**

WGS is now evidently capable of replacing traditional diagnostic procedures for tuberculosis in high-income settings. It offers clear benefits compared with traditional diagnostics, allowing rapid identification and control of outbreaks and minimising empirical treatment of patients through simultaneous first-line and second-line drug susceptibility prediction. Results from this study have led to a full feasibility study of use of WGS-based tuberculosis diagnostics in the UK, representing a paradigm shift in infectious disease diagnostics. Furthermore, as WGS technology continues to develop and portable systems become available, WGS will revolutionise diagnostics in low-income settings.

## Methods

### Study design

Eight participating laboratories in the UK, Ireland, Germany, France, and Canada processed all newly positive MGIT cultures from specimens submitted for mycobacterial testing on the examining physician's request between Sept 6, 2013, and April 14, 2014 (appendix p 19). We used no other selection criteria (except for the German centre where only the second positive primary culture from MTBC patients was available for processing). If patients had duplicate specimens—eg, from different body sites or at different times—we included them.

Because this study assessed service delivery methods without returning results for clinical management, research ethics committee approval was not required in the UK. Other centres obtained local ethics committee approval.

### Procedures

Routine diagnostic procedures at all centres included species identification (Hain GenoType MTBC/CM/AS), and for MTBC, MIRU-VNTR and culture with isoniazid, rifampicin, ethambutol, and pyrazinamide to establish drug susceptibility. Culture with streptomycin was also done in some centres. Subsequent culture of rifampicin-resistant isolates with fluoroquinolones and aminoglycosides was done to establish additional drug susceptibilities.

Each site prepared DNA and did WGS. We heat inactivated 1–2 mL MGIT culture aliquots at 95°C for 0·5–2 h, adhering to local protocols, and always retaining sufficient MGIT culture for routine diagnostic procedures to avoid compromising patient care. We isolated DNA as previously described (appendix);^20^ an in-house protocol was used in the Canadian centre. We prepared sequencing libraries for the MiSeq platform using a modified Nextera XT (Illumina) protocol (appendix) and sequenced pools of 11–15 MGIT samples plus *M tuberculosis* H37Rv or *BCG* DNA (positive control) with MiSeq version 2 2 × 150 bp paired-end read cartridges. We processed each sample once, repeating sequencing only for poor overall run performance. We deposited reads in the National Center for Biotechnology Information Short Read Archive (BioProject PRJNA268101 and PRJNA302362; appendix).

Staff doing WGS processing and analysis were masked to routine reference laboratory and clinical results. We shared MiSeq runs via Illumina BaseSpace and downloaded them at the Oxford centre for semi-automated analysis by a bespoke bioinformatics pipeline (appendix). A gene presence or absence algorithm identified mycobacterial species (using a catalogue of 169 sequenced mycobacterial strains). We mapped isolates identified as MTBC to the H37Rv reference genome (GenBank NC000962.2; Stampy version 1.0.22) and examined them for mutations deemed to confer phenotypic resistance to isoniazid, rifampicin, ethambutol, pyrazinamide, streptomycin, fluoroquinolones, or aminoglycosides on the basis of a published catalogue of high-confidence resistance-determining alleles (appendix).^6^ A minimum sequencing depth of five reads was needed to identify mutations; when we found more than one base at a single site, if the minority variant consisted of at least 10% of the total base calls and had a depth of at least five reads, we predicted a mixed phenotype. We identified cases compatible with transmission from a published database of 2191 UK MTBC sequences, representing all worldwide lineages, as previously described (using a threshold of 12 or fewer single-nucleotide polymorphisms (SNPs) on the basis of maximum diversity within different body sites and over time within a patient, and within household outbreaks; appendix).^13^

We estimated sequencing quality with several methods. For all specimens, we mapped the first 50 000 reads (Bowtie version 2.2.0) to the human genome (GRCh37/hg19) and nasal and mouth flora in the National Institutes of Health Human Microbiome Project. To verify that this sample was random, we selected 1% of reads using SAMtools 1.2 (SAMtools view –s option). We mapped these randomly selected reads using the same methods and yielded the same results (data not shown). We assessed the number of reads mapping to these human, nasal, and mouth databases, guanine-cytosine (GC) content (expected to be about 65% for mycobacteria), the number of reads available for analysis, the number of reads mapping to the reference genome, and reference genome coverage as predictors of accurate species identification using multivariable fractional polynomial logistic regression (Stata mfp; backwards elimination threshold p=0·05) in Stata 13.1, treating each specimen as an independent observation.^21^ We reported quality control data to the sequencing centre together with mycobacterial species, and for MTBC, drug susceptibility predictions and closest genomic match (appendix).

We gathered anonymised routine diagnostic data from local clinical laboratories and regional mycobacterial reference laboratories after WGS processing. All MTBC duplicate specimens were identified by the participating centres and analysis of drug resistance and outbreak incidence done with and without removal of duplicates (appendix). We did not identify duplicates for non-MTBC specimens. If routine and WGS results differed, we did additional quality checking and routine workflow assays; we did not repeat WGS. We calculated confidence intervals for WGS sensitivity, specificity, or accuracy compared with routine diagnostics in Stata 13.1 (Stata cii).

To assess the financial viability of WGS-based diagnostics, we did a microcosting analysis at a local clinical laboratory (John Radcliffe Hospital, Oxford, UK) and regional reference laboratory (Birmingham Heartlands Hospital Trusts, Birmingham, UK). We collected data using questionnaires based on standard operating procedures, expert consultations, and interviews with laboratory staff. Questionnaires were completed by clinical scientists doing mycobacterial processing and financial managers, who collected costs associated with staff time, error rates, equipment, and consumables (appendix). We obtained basic cost data (staff time, consumables, and equipment only) via interview with clinical scientists for second-line phenotypic DST (done at the National Mycobacterial Reference Laboratory, London, UK). We annualised costs using the throughput of the Birmingham regional reference laboratory for 2014.

### Role of the funding source

The funder of the study had no role in study design, data collection, data analysis, data interpretation, or writing of the report. The corresponding author had full access to all the data in the study and had final responsibility for the decision to submit for publication.

## Results

Each participating site collected positive MGIT samples for between 9 and 158 days (appendix p 19). 27 (21%) of 127 MTBC patients provided two to six specimens. Median time from positivity to inactivation was 4 days (IQR 1–5; 96 recorded). Median read depth, based on read length and number of reads mapping to the reference genome, was 73 (IQR 36–99; see appendix for alternative read-depth metrics).

356 MGIT specimens were submitted. 345 (97%) were identified to species or complex by routine diagnostic workflows (table 1). In 326 (94%) cases, both Hain and WGS assays identified a single species, of which three (1%) were discordant. Two species were identified in nine (3%) of cases, of which eight (89%) were discordant for one of the species. WGS predictions were concordant with routine results in 322 (93% [95% CI 90–96]) of 345 specimens (including duplicate specimens). Hain and WGS assays identified MTBC in 168 (52%) of 322 concordant specimens. Of the discordant isolates, three (13%) of 23 were MTBC cases identified by the reference laboratory alone, three (13%) were MTBC cases identified by WGS alone, and two (9%) were identified in a co-infection by either WGS or the reference laboratory (but not both). In a further six (26%) MTBC cases (identified by the reference laboratory), WGS failed. Overall, MTBC was identified with 95% (95% CI 91–98) sensitivity and 98% (95–100) specificity (including duplicate specimens). Causes of discordant results were mixed or contaminated samples and poor quality sequencing. Failure to identify MTBC increased significantly and independently if GC content fell below 50% (showing low-GC non-mycobacterial DNA contamination) or if the total number of sequencing reads fell below 1 million (appendix p 16; p<0·005).

For 168 MTBC specimens identified by WGS and routine diagnosis, 628 (93% [95% CI 91–95]) of 672 WGS-based first-line drug susceptibility predictions were concordant with reference laboratory DST. After deduplication, 467 (92% [89–94]) of 508 predictions across 127 specimens were concordant. Overall, WGS resistance prediction failed on 63 occasions across 15 specimens. Eight (53%) specimens failed all predictions, four (27%) failed aminoglycosides only, two (13%) failed rifampicin only, and one (7%) failed pyrazinamide only. When WGS prediction failed, all but one DST result was sensitive (one DST also failed). WGS made an additional 434 (mainly second-line) predictions when DST was not done, including four specimens with monoresistance to second-line drugs (appendix). In 13 cases (across 11 specimens and four drugs), resistance-conferring mutations and wild-type alleles occurred as a mixture, preventing phenotypic predictions. We noted the highest number of mixtures (7 [4%] of 168 specimens) in genes conferring resistance to aminoglycosides (table 2). Eight WGS predictions (across six specimens and six drugs) were discordant with DST. Of seven phenotypically resistant specimens with no resistance-conferring mutations in the catalogue, six (86%) contained unclassified variants in the relevant genes (appendix). Of the 127 deduplicated specimens, WGS reported 22 (17%) incidences of drug-resistant MTBC, of which five (23%) were *M bovis* (monoresistant to pyrazinamide). Four (3%) of 127 patients were infected with multidrug-resistant tuberculosis.

Pairwise SNP distances between 16 UK-sourced H37Rv-positive controls were all zero; one, from different starting material, was eight SNPs or fewer from other replicates. 68 (40%) of 168 MTBC specimens were 12 SNPs or fewer from at least one other specimen in the available UK database or previously sequenced in this study; however, 33 (49%) of these specimens (15 patients) were linked only to another specimen from the same patient.^13^ In the 127 patients with MTBC, the median pairwise distance to the next nearest patient was 113 (IQR 48–173). 22 (17%) patients with MTBC (19 [86%] UK and three [14%] non-UK; 35 specimens) were linked to different patients. This number included five (23%) patients (four [80%] UK; one [20%] non-UK) with vaccine-strain BCG and two (9%) patients linked only to each other (same non-UK centre). In total, 15 (16% [95% CI 10–26]) of 91 UK patients studied were linked to nine outbreak clusters in the UK database; including two (13%) patients unexpectedly linked to an outbreak cluster (subsequently confirmed epidemiologically; pilot cluster 6; appendix) and two (13%) from different UK regions linked to an isoniazid-resistant cluster in a third region (subsequently confirmed epidemiologically and via MIRU-VNTR; appendix). Eight (89%) of nine outbreak clusters were already being investigated by local health protection teams, with well established epidemiological links supported by MIRU-VNTR data (available for eight [53%] of 15 UK study patients linked to these pre-existing clusters). The remaining cluster involved patients infected with multidrug-resistant tuberculosis. In this case, WGS provided the first diagnosis and outbreak alert (panel).

**Figure 2:**
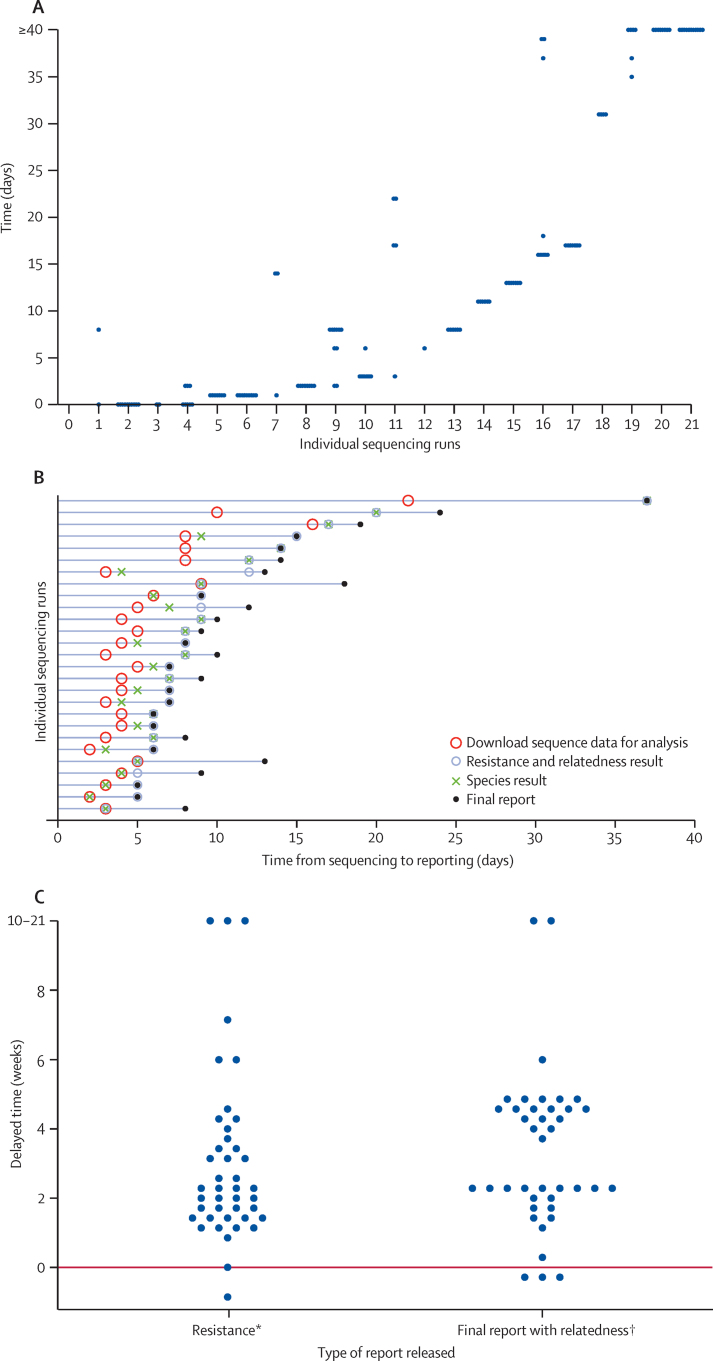
Timings for whole-genome sequencing and routine methods (A) Time taken to extract DNA from individual Mycobacteria Growth Indicator Tube aliquots and do each sequencing run (one point per isolate); (B) time from the sequencing run finishing to sharing of data to generation of reports; (C) time taken for reference laboratory reports of resistance and relatedness to be generated compared with whole-genome sequencing (WGS) reports (one point per isolate). Reference laboratory timings calculated from the sending of samples to the reference laboratory; adjusted WGS timings calculated with an assumption of 2 days (laboratory processing time) between the date samples were sent to the reference laboratory and the date sequencing was done (minimising batch effects); data immediately shared. Points below 0 show that reference laboratory reports were generated faster than WGS reports were. *Receiving of resistance report from reference laboratory compared with generation of resistance prediction by WGS. †Receiving of final report, including mycobacterial interspersed repetitive unit-variable-number tandem repeat data, from reference laboratory compared with final WGS reports being generated (including relatedness).

Unadjusted median time from MGIT positivity to DST reporting was 25 days (IQR 14–32), whereas for final reports, including MIRU-VNTR genotype reporting, it was 31 days (IQR 21–44); similarly, full WGS-based reports were available in 31 days (IQR 21–60). WGS processing delays were driven by sample batching for sequencing and delays in sharing sequencing data (figure 2). Additionally, we adhered to a 5 day working week for WGS-based processing, rather than the 7 day working week in clinical laboratories. After WGS sharing, we generated full diagnostic reports in a median of 5 days (IQR 3–7). The time delay from sample batching would be minimised in high-throughput laboratories. To estimate the potential speed of WGS-based diagnosis, we compared the recorded times from 2 days before sequencing (to allow for sample preparation and 7 day working weeks) to WGS report generation with reference laboratory reporting times (using the date that specimens were sent to the reference laboratory as the starting point). We found that reference laboratory reports were generated a median of 15 days (IQR 9–25) slower than we could produce WGS reports for drug resistance (median 24 days [IQR 20–33] *vs* 8 days [6–9]) and 21 days (14–32) slower for relatedness (32 days [22–42] *vs* 9 days [6–10]; figure 2).

The cost of WGS-based diagnosis, routine diagnostic costs for a non-tuberculous mycobacteria (NTM; culture and species identification only), costs for a fully sensitive MTBC (culture, species identification, MIRU-VNTR, and first-line DST), and costs for a drug-resistant MTBC (culture, species identification, MIRU-VNTR, first-line and second-line DST if resistant to rifampicin) are shown in table 3 (detailed breakdown in appendix). Consumables were the main cost driver for all processes other than for DST, for which staff costs dominated.

Costs (calculated with reported throughput for 2014) for routine diagnostic workflows were £518 per culture-positive specimen, consisting of MGIT culture for all samples received, species identification for culture-positive specimens, and MIRU-VNTR and DST for MTBC-positive specimens. For WGS-based diagnosis, consisting of MGIT culture for all samples received and WGS for culture-positive specimens, the per-culture-positive specimen cost would be £481, which is 7% cheaper than are routine diagnostics. To do DST as per present workflows alongside WGS would cost £540 per culture-positive specimen, which is 4% more expensive than are routine diagnostics (table 3). Increasing sample throughput decreased costs overall (table 3, appendix). Variation in sequencing batch size, throughput, error rates, equipment, consumables, and overhead costs could alter overall WGS costs by up to 17% (appendix).

## Discussion

In this prospective, multicentre, international pilot study, we assessed the real-time performance and cost of WGS for laboratory diagnosis of mycobacterial infection. With use of prototype software and only one sequencing attempt, 93% of mycobacteria were concordant with routine laboratory complex or species-level identification and 93% of susceptibility predictions for MTBC isolates were concordant with DST. The same sequencing data linked 16% of UK patients to an outbreak, identified an inter-regional cluster of isoniazid-resistant MTBC, and discovered transmission of multidrug-resistant tuberculosis, substantially hastening diagnosis and appropriate treatment of one patient. Generation of full diagnostic information by WGS was 7% cheaper than by routine methods.

This study provides proof-of-principle in high-income countries that local sample collection and sequencing with centralised analysis can be applied on an international scale. The median of 5 days between WGS data sharing and full diagnostic report availability compares favourably with present diagnostic workflows.^5^, ^12^ The resistotyping and nearest-neighbour algorithms are rapid and scalable, retaining the resolution of whole-genome analysis. Our workflow does not depend on the specific algorithms used, which could easily be refined and improved. Consequently, Public Health England is currently assessing the suitability of WGS from early-positive MGIT cultures to replace routine clinical tuberculosis processing.^22^

For species identification, Hain assays are capable of detecting 40 NTM and *M tuberculosis* species or complexes. The sequenced catalogue of 169 mycobacterial type strains used here provided broad diagnostic capacity. Although the gene presence or absence algorithm could not distinguish between different complex members, for MTBC, this issue was resolved by phylogenetic analysis after reference genome mapping. This approach could be extended to other species' complexes.

WGS drug susceptibility predictions were highly concordant with the reference standard, simultaneously predicting first-line and second-line susceptibilities at no additional cost, despite incomplete knowledge of genotype–phenotype relations. Overall, the 17% incidence of drug resistance and 3% incidence of multidrug-resistant tuberculosis, as assessed by WGS, was well below occurrence in high-incidence settings.^10^ Of eight discrepant results, four had unclassified variants not in our prespecified catalogue, but with evidence of association with drug resistance in the wider scientific literature. Investigations have provided robust, evidence-based resistance prediction catalogues for use in future investigations and algorithms for the iterative addition of new or rare mutations to the prediction catalogue, with phenotypic support.^6^, ^8^, ^10^, ^14^ No previously described mutations could explain phenotypic resistance for the remaining specimens; with only one sequencing attempt per isolate, these data could not be verified. Drug resistance might also be mediated by post-transcriptional or post-translational protein modifications and efflux pump activation.^23^, ^24^, ^25^ Such resistance mechanisms remain little studied and should be investigated further as genotype–phenotype comparisons continue. However, DST methods for drugs other than isoniazid and rifampicin are also imperfect.^26^, ^27^

Minority variants complicating phenotypic predictions were present in 12 isolates. These variants might be due to emerging resistance or mixed infection within the patient, sample contamination with nasopharyngeal flora, or cross-contamination. We noted more mixed calls (4%) in the 16S gene (*rrs)* conferring aminoglycoside resistance than in the other resistance-conferring genes examined, potentially due to similar reads from other bacterial species mapping to the H37Rv reference. Because MGITs are inoculated with primary clinical samples, removal of other bacterial DNA, and subsequent resistance prediction where removal has been incomplete, pose challenges.^20^ Despite this challenge, overall sensitivity was similar to that reported from other available drug susceptibility prediction tools and with use of pure-culture samples.^6^, ^8^ Throughout this investigation, WGS results were provided together with sequence quality feedback to allow laboratory staff to improve interpretation and practice. Other methods provide a small amount of data quality feedback and drug resistance identification, but do not provide species or outbreak analysis.^6^, ^8^ Although WGS was not repeated in this study, retesting isolates when species identification, DST, or MIRU-VNTR deliver questionable results is routine in diagnostic laboratories, and such a system will be implemented for WGS. As more specimens are sequenced than at present, robust algorithms to identify isolates for resequencing will be developed to prevent inaccurate results being reported and improve WGS sensitivity and specificity.

16% of sequenced UK MTBC isolates were linked to one of nine UK outbreak clusters, including one spanning three regions. Large geographical distances separating genetically related isolates have been previously reported,^10^ with the authors concluding that low genetic divergence might not always represent transmission or that casual contact might be important in MTBC transmission. Application of WGS on a wide scale, as shown by the US Food and Drug Administration,^28^ will only serve to increase the number of links detected between patients and outbreaks. Most surprisingly during this investigation, WGS diagnosed multidrug-resistant tuberculosis in one patient, only subsequently confirmed by the reference laboratory. This diagnosis directly affected the individual patient's care and reduced onward transmission risk. Identification of a second patient with genetically identical multidrug-resistant tuberculosis shows the need to rapidly identify infected patients to minimise the risk of transmission.

An often cited obstacle to clinical implementation of WGS is cost.^29^, ^30^, ^31^ For the laboratories and workflows costed here, high WGS costs for NTM diagnosis were outweighed by savings made in MTBC diagnosis, leading to an overall saving of 7% per year for a reference centre. If present DST workflows are continued alongside WGS, costs would be 4% greater per year than with present workflows alone. However, these additional costs would be mitigated by replacement of any molecular DST done alongside phenotypic DST with WGS. The cost-effectiveness of replacement of phenotypic with other rapid genotypic assays in terms of patient care has already been shown^31^ and is probably similar for WGS. The decentralised-sequencing, centralised-analysis model used in this study minimises computational and technical support costs. WGS costs could fall further when implemented diagnostically, which will need less skilled staff than at present. However, availability of skilled staff is likely to remain a key limitation of adoption of WGS in low-income settings. Fully automated analysis and reporting and strategic placement of benchtop sequencers would also reduce diagnostic delays, as reported in this study. Furthermore, progress in development of direct-from-sample and point-of-care WGS is continuing, and, combined with the analysis algorithms shown, will revolutionise MTBC diagnosis.^31^, ^32^

WGS allows simultaneous prediction of mycobacterial species, first-line and second-line drug resistance, the ability to monitor emergence of new resistance mechanisms, and high-resolution outbreak monitoring on a timescale weeks faster than with traditional diagnostics. Coupled with public health interventions, WGS will transform MTBC patient care and disease control and has the potential to transform diagnosis of other infectious diseases.^5^ Furthermore, our cost estimates have shown that WGS-based diagnostics will provide value for money, demonstrating how WGS can replace mycobacterial diagnostic workflows from positive MGIT culture in high-income countries.

## Figures and Tables

**Figure 1:**
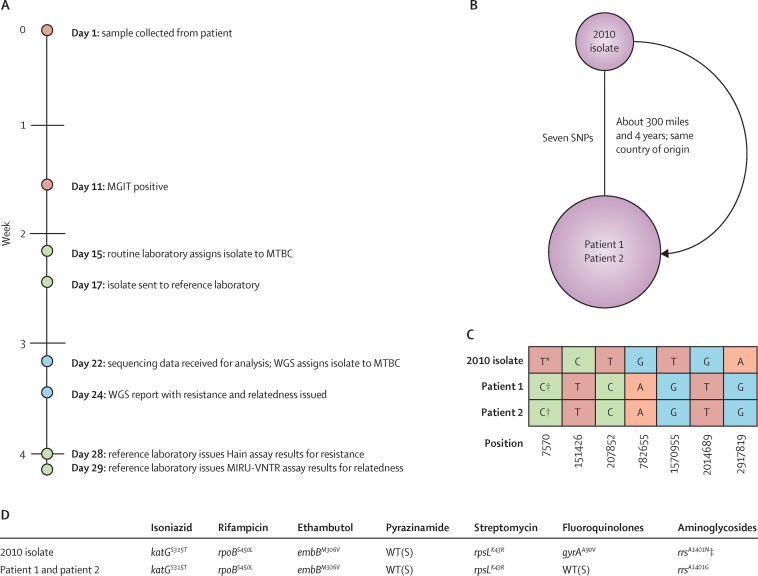
Details of pilot study cluster 7 (A) Timeline of the full WGS-based, routine-based, and reference laboratory-based diagnosis for patient 1 (red circles represent processes needed by both routine or reference and WGS processes, green circles represent routine or reference processes, and blue circles represent WGS processes); (B) the genomic relation between cluster isolates as established by WGS nearest neighbours; (C) detail of the seven SNP differences between cluster isolates; (D) detail of the resistance-conferring mutations identified in the 2010 isolate and the two study patients. A=adenine. C=cytosine. G=guanine. MIRU-VNTR=mycobacterial interspersed repetitive unit-variable-number tandem repeat. MGIT=Mycobacteria Growth Indicator Tube. MTBC=*Mycobacterium tuberculosis* complex. SNP=single-nucleotide polymorphism. T=thymine. WGS=whole-genome sequencing. WT(S)=wild-type (susceptible). *Mutation conferring resistance to fluoroquinolones. †Wild-type (sensitive). ‡Heterozygous at this position (four wild-type base calls A *vs* 29 resistance-conferring variant base calls G).

**Table 1:** Concordance between single WGS and routine laboratory methods for mycobacterial speciation

		**n**
Routine methods and WGS identification failed		9
Routine methods failed		2*
Identified by routine methods		345 (100%)
Concordant		322 (93%)
	*M tuberculosis* complex	157 (46%)
	*M avium* complex	71 (21%)
	*M abscessus* complex	39 (11%)
	*M gordonae*	18 (5%)
	*M xenopi*	11 (3%)
	*M tuberculosis* complex (BCG)	8 (2%)
	*M kansasii*	6 (2%)
	*M malmoense*	3 (1%)
	*M fortuitum*	2 (1%)
	*M szulgai*	2 (1%)
	*M tuberculosis* complex (*M africanum*)	2 (1%)
	*M celatum*	1 (<1%)
	*M lentiflavum*	1 (<1%)
	*M tuberculosis* complex and *M avium* complex	1 (<1%)
Part concordant		10 (3%)
WGS gained one species		5 (1%)†
WGS missed one species		3 (1%)‡
WGS identified related species		1 (<1%)§
WGS identified subspecies		1 (<1%)¶
Discordant		3 (1%)‖
WGS failed		10 (3%)

Data in parentheses are % of specimens identified by routine methods. *M*=*Mycobacterium*. WGS=whole-genome sequencing.

* WGS identified *M avium* complex (good quality WGS; 32 [97%] of 33 genes identified) and *M kumamotonense* (poor quality WGS; 58 [58%] of 100 genes identified).

† One routine *M tuberculosis* complex (MTBC): WGS identified MTBC plus *M avium* complex (retesting not possible); three routine *M avium* complexes: WGS identified MTBC plus *M avium* complexes (two retesting supported routine; one supported WGS); one routine *M abscessus* complex: WGS identified *M abscessus* plus *M avium* complex (retesting supported routine); appendix.

‡ One routine MTBC plus *M avium* complex: WGS identified MTBC (retesting supported WGS); two routine MTBC plus *M avium* complex: WGS *M avium* complex (one poor quality WGS; two [6%] of 33 genes identified; one unable to retest); appendix.

§ One undescribed mycobacterial species similar to *M avium*: WGS identified *M avium* complex (appendix).

¶ One routine *M fortuitum*: WGS identified *M fortuitum*-*acetamidolyticum* (poor quality WGS; 33 [33%] of 100 genes identified); appendix.

‖ One routine *M kansasii*: WGS identified *M avium* complex (routine testing subsequently confirmed *M avium* complex); one routine *M avium* complex: WGS *M scrofulaceum* (poor quality WGS; one [1%] of 72 genes identified); one routine MTBC: WGS identified *M abscessus* complex (good quality WGS; 38 [95%] of 40 genes identified; unable to retest specimen); appendix.

**Table 2:** Whole-genome sequencing resistance predictions for *Mycobacterium tuberculosis* complex specimens compared with phenotypic DST

		**DST successful: resistant**				**DST successful: sensitive**				**DST failed**				**DST not attempted**			
		Resistant	Sensitive	Mixed*	Failed†	Resistant	Sensitive	Mixed*	Failed†	Resistant	Sensitive	Mixed*	Failed†‡	Resistant	Sensitive	Mixed*	Failed†
Total across drugs and drug classes§		40 (100%)/19 (100%)	7 (100%)/6 (100%)	1 (100%)/1 (100%)	0	1 (100%)/1 (100%)	618 (100%)/120 (100%)	5 (100%)/4 (100%)	31 (100%)/8 (100%)	0	1 (100%)/1 (100%)	0	4 (100%)/0	6 (100%)/4 (100%)	427 (100%)/118 (100%)	7 (100%)/6 (100%)	28 (100%)/10 (100%)
First-line drugs																	
	Isoniazid	13 (33%)/11 (56%)	2 (29%)/2 (33%)	1 (100%)/1 (100%)	0	0	143 (23%)/105 (88%)	0	7 (23%)/7 (88%)	0	1 (100%)/1 (100%)	0	1 (25%)/0	0	0	0	0
	Rifampicin	5 (13%)/4 (21%)	1 (14%)/1 (17%)	0	0	0	148 (24%)/111 (93%)	4 (80%)/3 (75%)	9 (29%)/8 (100%)	0	0	0	1 (25%)/0	0	0	0	0
	Ethambutol	5 (13%)/4 (21%)	1 (14%)/1 (17%)	0	0	1 (100%)/1 (100%)	153 (25%)/114 (95%)	0	7 (23%)/7 (88%)	0	0	0	1 (25%)/0	0	0	0	0
	Pyrazinamide	8 (20%)/5 (26%)	1 (14%)/1 (17%)	0	0	0	149 (24%)/113 (94%)	1 (20%)/1 (25%)	8 (26%)/7 (88%)	0	0	0	1 (25%)/0	0	0	0	0
Second-line drugs																	
	Streptomycin	5 (13%)/3 (16%)	1 (14%)/1 (17%)	0	0	0	14 (2%)/12 (10%)	0	0	0	0	0	0	2 (33%)/1 (25%)	138 (32%)/103 (87%)	0	8 (29%)/7 (70%)
	Fluoroquinolones	3 (8%)/3 (16%)	1 (14%)/1 (17%)	0	0	0	6 (1%)/5 (4%)	0	0	0	0	0	0	2 (33%)/2 (50%)	148 (35%)/109 (92%)	0	8 (29%)/7 (70%)
	Aminoglycosides	1 (3%)/1 (5%)	0	0	0	0	5 (1%)/4 (3%)	0	0	0	0	0	0	2 (33%)/1 (25%)	141 (33%)/105 (89%)	7 (100%)/6 (100%)	12 (43%)/10 (100%)

Data are number of specimens (%)/number of patients (%). DST=drug susceptibility testing.

* Resistant and sensitive.

† Failed whole-genome sequencing prediction (insufficient sequencing data to predict drug resistance; each specimen sequenced only once).

‡ There are zero patients because these samples have been removed during removal of duplicate specimens.

§ The numbers of patients do not add to the totals because patients can be counted in more than one category.

**Table 3:** Total cost per sample by process, accounting for error rates

		Throughput in 2014 (n)*	**Total per sample in 2014 (£)**	**10% fewer samples per year (£)**	**10% more samples per year (£)**
**WGS and routine clinical workflows**					
MGIT culture		15 265	52·39	52·90	51·97
Cepheid Xpert MTB/RIF		617	99·66	102·35	97·44
**WGS workflow only**					
WGS		2207	118·55	120·16	117·26
**Routine clinical workflows only**					
Identification assays		2207	55·05	55·28	54·87
	Hain MTBC	866	..	..	..
	Hain CM/AS	1341	..	..	..
MIRU-VNTR		866	107·75	110·89	105·18
First-line DST		866	135·47	137·12	134·13
Limited second-line DST†		62	93·01	93·24	92·83
Second-line DST‡		62	101·27	104·24	98·86
**WGS workflow scenarios**					
MGIT culture and WGS		..	170·94	173·06	169·23
MGIT culture and WGS and first-line DST		..	306·41	310·18	303·36
MGIT culture and WGS and first-line DST and full second-line DST		..	500·68	507·66	495·05
**Routine clinical workflow scenarios**					
Culture and identification assays		..	107·44	108·18	106·84
Culture and identification assays and MIRU-VNTR and first-line DST		..	350·66	356·19	346·15
Culture and identification assays and MIRU-VNTR and first-line DST and full second-line DST		..	544·93	553·69	537·84
**Total workflow costs**					
WGS-based diagnostics		..	480·91	486·01	476·75
WGS-based diagnostics and first-line and full second-line DST		..	539·53	545·37	534·73
Routine clinical workflow-based diagnostics		..	518·31	524·00	513·64

Error rates reported in this study: 1% microscopy, 2% MGIT culture, 10% Cepheid Xpert MTB/RIF, <1% species identification (Hain ID), 13% DNA extraction for WGS, 4% WGS, 1% WGS data analysis, 10% MIRU-VNTR, and <1% DST. WGS=whole-genome sequencing. MGIT=Mycobacteria Growth Indicator Tube. MIRU-VNTR=mycobacterial interspersed repetitive unit-variable-number tandem repeat. DST=drug susceptibility testing.

* Number reported in the Birmingham reference laboratory (Birmingham, UK) for 2014. Negative tests not counted for Hain GenoType MTBC (Hain Lifescience, Nehren, Germany), Hain GenoType Mycobacterium CM/AS, second-line DST, Hain GenoType MTBDR*plus*, or Hain GenoType MTBDR*sl*.

† Done at the Birmingham clinical laboratory.

‡ Done at a second reference laboratory (London, UK). Based on staff time, consumables, and equipment only.

## Panel Early multidrug-resistant tuberculosis diagnosis and outbreak discovery

Two patients were linked to a previously sequenced extremely drug-resistant tuberculosis case to form a new cluster (figure 1). The first case, for which sequencing and analysis was completed a week after Mycobacteria Growth Indicator Tube positivity, was seven single-nucleotide polymorphisms from a 2010 isolate from a patient residing about 300 miles away.^13^ The study specimen was predicted to be resistant to isoniazid, rifampicin, and aminoglycosides, whereas the 2010 isolate was phenotypically and genotypically resistant to the same drugs and to fluoroquinolones (with *gyrA*^A90^ mutation). Because the study specimen had yet to be processed by the reference laboratory, whole-genome sequencing (WGS) provided the first multidrug-resistant tuberculosis alert to the participating centre. This patient, with smear-positive pulmonary tuberculosis, was receiving first-line treatment in the community while awaiting laboratory results. Prompted by WGS, urgent Hain MTBDR*plus* and MTBDR*sl* assays were done by the reference laboratory, confirming WGS drug resistance predictions. Mycobacterial interspersed repetitive unit-variable-number tandem repeat was identical across the 2010 and study isolates, confirming the genetic relation. The patient was admitted to hospital for appropriate treatment on the basis of reference laboratory Hain results. Epidemiological investigation showed that both patients originated from the same European country. 2 months later, a second study specimen from the same centre was zero SNPs from the first specimen, with an identical drug resistance profile, consistent with direct transmission.^13^ Mycobacterial interspersed repetitive unit-variable-number tandem repeat again confirmed the genetic relation, although no epidemiological links between these patients have yet been identified.
