# Supplementary material for: Rapid, comprehensive, and affordable mycobacterial diagnosis with whole-genome sequencing: a prospective study
Source: Lancet Respir Med. 2016 Jan;4(1):49–58. doi: 10.1016/S2213-2600(15)00466-X (PMC4698465; doi:10.1016/S2213-2600(15)00466-X)
Supplement: Supplementary appendix [file mmc1.pdf]

THE LANCET
Respiratory Medicine

Supplementary appendix

This appendix formed part of the original submission and has been peer reviewed. We post it as supplied by the authors.

Supplement to: Pankhurst LJ, del Ojo Elias C, Votintseva AA, et al, for the COMPASS-TB Study Group. Rapid, comprehensive, and affordable mycobacterial diagnosis with whole-genome sequencing: a prospective study. *Lancet Respir Med* 2015; published online Dec 3. http://dx.doi.org/10.1016/S2213-2600(15)00466-X.

Supplementary appendix

Table of Contents

COMPASS-TB STUDY GROUP 2

Methods 5

Figure S1: 9

Figure S2: 10

Figure S3: 11

Figure S4: 12

Figure S5: 13

Figure S6: 14

Figure S7: 15

Figure S8: 16

Figure S9: 17

Figure S10: 18

Table S1 19

Table S2 20

Table S3: 24

Table S4: 25

Table S5: 26

Table S6: 27

Table S7: 31

Table S8: 32

Table S9: 33

Table S10: 34

Table S11: 35

COMPASS-TB STUDY GROUP

Study design committee
Derrick W Crook (Chair), Ibrahim Abubakar, Laura Fay Anderson, Helen Barker, Jennifer Gardy, Jonathan Green, Saheer Gharbia, Martin Llewelyn, John Magee, Philip Monk, Stefan Niemann, John Paul, Timothy E A Peto, Thomas R Rogers, E Grace Smith, Philip Supply, Helen Lucy Thomas, A Sarah Walker, Mark H Wilcox, David Wyllie, Maria Zambon.

Study co-ordination committee
Derrick W Crook (Chair), Margaret M Fitzgibbon, Saheer Gharbia, Stefan Niemann, Louise J Pankhurst, John Paul, Mabel Rodrigues, Thomas Rogers, E Grace Smith, Philip Supply, Patrick Tang, Mark H Wilcox

Independent steering committee
Gail Bolan (Chair), Jane Binyon, Peter Donnelly, Christine McCartney, Arthur Reingold, Michael Sharland, Tom Tsatsonis

Specimen collection, preparation and sequencing
Caroline Allix-Béguec , Kevin Cole, Tatiana Collins, Guy Delcroix, Jason Evans, Margaret M Fitzgibbon, Deborah M Gascoyne-Binzi, Cyril Gaudin, Ximena Gonzalo, Thomas A Kohl, Clare Kong, Nadine Lemaitre, Mathilde Mairey, Marcus Morgan, Robert O'Hara, Louise J Pankhurst, Margot Patin, Elvira Richter, Emma Roycroft, Mihanta Ramaroson, Antonina A Votintseva, Li Xu

Data analysis group
Timothy E A Peto, Louise J Pankhurst, Antonina A Votintseva, A Sarah Walker, Timothy M Walker, David Wyllie

Bioinformatics group
Tanya Golubchik, Vasiliki Kostiou, Carlos del Ojo Elias, Dilrini de Silva

Health economics group
Jilles M Fermont, Priti Rathod, E Grace Smith, Sarah Wordsworth

Informatics group
Milind V Acharya, Charles Crichton, Jim Davies, Laura Madrid Marquez, Carlos del Ojo Elias, James Welch

Manuscript writing and editing committee
Ibrahim Abubakar, Laura Fay Anderson, Derrick W Crook, Francis Drobniewski, Jennifer Gardy, Louise J Pankhurst, John Paul, Timothy E A Peto, Thomas R Rogers, Philip Supply, Helen Lucy Thomas, Antonina A Votintseva, A Sarah Walker, Timothy M Walker, Sarah Wordsworth

INVESTIGATORS BY AFFILIATION

Brighton and Sussex University Hospitals NHS Trust, Brighton, UK
Lead Investigator: John Paul
Martin Llewelyn, Kevin Cole

British Columbia Centre for Disease Control, Vancouver, Canada
Lead Investigator: Patrick Tang
Jennifer Gardy, Clare Kong, Mabel Rodrigues

Department of Clinical Microbiology Trinity College Dublin, and Irish Mycobacteria Reference Laboratory, St James's Hospital, Dublin, Ireland
Lead Investigator: Thomas R Rogers
Margaret M Fitzgibbon, Emma Roycroft

Molecular Mycobacteriology, Forschungszentrum Borstel, Leibniz-Zentrum für Medizin und Biowissenschaften, Borstel, Germany
Lead Investigator: Stefan Niemann††
Thomas A Kohl, Elvira Richter

Genoscreen, Lille, France
Lead Investigator: Philip Supply*
Caroline Allix-Béguec, Cyril Gaudin, Mathilde Mairey, Mihanta Ramaroson

Leeds Teaching Hospitals NHS Trust, Leeds, UK
Lead Investigator: Mark H Wilcox MD
Deborah M Gascoyne-Binzi, Robert O'Hara

Oxford University Hospitals NHS Trust, Oxford, UK
Marcus Morgan

Public Health England Regional Centre for Mycobacteriology, Birmingham Heartlands Foundation NHS Trust, Birmingham, UK
Lead Investigator: E Grace Smith FRCPath
Tatiana Collins, Jason Evans, Priti Rathod, Li Xu

Centre Hospitalier Universitaire, Lille, France
Lead Investigator: Nadine Lemaitre*
Guy Delcroix, Margot Patin, Philip Supply*

University of Oxford, Oxford, UK
Lead Investigator: Derrick W Crook
Milind V Acharya, Charles Crichton, Jim Davies, Jilles M Fermont, Tanya Golubchik, Vasiliki Kostiou, Laura Madrid Marquez, Carlos del Ojo Elias, Louise J Pankhurst, Timothy E A Peto, Dilrini de Silva, Antonina A Votintseva, A Sarah Walker, Timothy M Walker, Sarah Wordsworth, David Wyllie

Public Health England National Mycobacterial Reference Laboratory, London, UK

Lead investigator: Francis Drobniewski ** and Ximena Gonzalo**

Public Health England Epidemiological Units, UK
Ibrahim Abubakar, Laura Fay Anderson, Saheer Gharbia, Jonathan Green, John Magee, Helen Lucy Thomas, Maria Zambon

††Also affiliated to:
German Center for Infection Research (DZIF), Borstel, Germany

***Also affiliated to:**
Université de Lille, CNRS, Inserm, Institut Pasteur de Lille, U1019 - UMR 8204 - Centre d'Infection et d'Immunité de Lille, all in Lille, France.

** **Also affiliated to:**
Department of Infectious Diseases, Imperial College, London, UK

Methods

Sample collection

All newly-positive MGIT cultures were collected and processed for this investigation, including duplicate specimens from the same patient. All specimens were collected between September 2013 and April 2014 (Table S1). No other selection criteria were imposed (except Borstel where only the second positive primary culture from MTBC patients was available for processing).

DNA extraction

Inactivated BACTEC™ MGIT™ (Beckton Dickinson, USA) aliquots (1-2 mL) were pelleted through centrifugation at 16100 rcf for 15 minutes. Supernatant was removed and the pellet re-suspended in 1 mL sterile saline before centrifugation at 16100 rcf for 15 minutes to re-pellet the aliquot, and removal of the supernatant. The pellet was then re-suspended in 700 µL of molecular grade water and the suspension mechanically disrupted in Lysing Matrix B (MP Biomedicals, USA) using the FastPrep-24 tissue homogeniser (MP Biomedicals, USA) with three cycles at 6 m/s for 40s. Following disruption the aliquot was centrifuged at 16100 rcf for 10 minutes, and 450 µL of supernatant transferred to a new 1·5 mL tube.

DNA was isolated through precipitation in the presence of 1:10 volumes of 3 M sodium acetate (45 µL) and 1:1 volumes of ice-cold ethanol (minimum 96%; 1 mL) and incubated at -20°C for one hour. The DNA was pelleted by centrifugation at 16100 rcf for 15 minutes. Supernatant was removed, and the DNA pellet washed twice with 70% ethanol before complete removal of the supernatant and air drying at room temperature for 10-15 minutes. The DNA pellet was re-suspended in 50 µL 1x Tris Ethylenediaminetetraacetic acid (TE) buffer at 55°C. 45 µL of the final supernatant was cleaned using Solid Phase Reversible Immobilisation (SPRI) beads (AMPure XP, Beckman Coulter, USA) in a volume of 1·8x beads to eluate (81 µL). Following manufacturer's protocols the bead pellet was washed twice with 70% ethanol and dried at room temperature for 10-15 minutes. DNA was eluted from the SPRI beads in 26 µL 1x TE buffer with 25 µL transferred to a new 1·5 mL tube and stored at -20°C.

Sequencing

Samples were normalised to 0·2 ng/µL following quantitation using the Qubit dsDNA High Sensitivity kit on the Qubit 2·0 Fluorometer (LifeTechnologies, USA). Sequencing libraries were prepared for MiSeq (Illumina, USA) sequencing using the Nextera XT protocol (Illumina, USA, Part #15031942 rev. C, October 2012). Manufacturer's instructions were followed with the following modifications: limited-cycle PCR amplification program was extended from 12 to 15 cycles, and libraries were manually normalised to 2-10 nM based on DNA concentration, or DNA concentration and average library size as measured by the Qubit dsDNA High Sensitivity kit on the Qubit 2·0 Fluorometer and the D1K High Sensitivity Screentape on the 2200 TapeStation (Agilent Technologies, USA). Libraries were pooled in equal volume, denatured according to the manufacturer's protocols and diluted to a sequencing concentration of up to 20 pM. Finally, 12·5 pM PhiX (Illumina, USA) was added at 1% of the loading volume.

Sequence processing

Sequence processing was blinded to clinical information and routine laboratory results. Completed sequencing runs were shared via Illumina BaseSpace and data downloaded to Oxford for semi-automated analysis by a bespoke bioinformatic pipeline (Figure S2). Reads were deposited in the

National Center for Biotechnology Information Short Read Archive (BioProject PRJNA268101, BioSample accession numbers SAMN03225300 to SAMN03225373 and SAMN03225375 to SAMN03225393; and all BioSamples in BioProject PRJNA302362).

Firstly, species were identified for each isolate using a gene presence/absence algorithm, developed as follows. The whole genome sequences of 169 commercially available mycobacterium type species were gathered; either through sequencing and assembly with Velvet v1·0·18 or NCBI. Using the assembled genomes, genes were annotated or predicted (Prokka v1·8) and clustered. Unique representatives of clusters were identified using cd-hit (4·5·4) and used to construct a mycobacterium pangenome. Raw reads from the 169 isolates were then mapped against the pangenome (BWA v 0·7·5a) to detect genes that were present only in one species. Those genes were used to generate a catalogue of unique genes for each mycobacterial species and/or cluster. Isolates sequenced throughout this investigation were mapped (Bowtie2 v2·0·0-beta7) to this catalogue of unique genes to identify species. A minimum read-depth of 5 and coverage of 80% was required to affirm the presence of a gene.

If the sequenced isolate was identified as belonging to MTBC, it was subsequently mapped to the H37Rv (GenBank NC000962·2) reference genome with Stampy (v1·0·22; mapped files available on request). Self-self BLAST was used to define repetitive regions, which were masked. Nucleotide calls were made with SAMtools mpileup (v0·1·08) and required a minimum depth of five reads with at least one read in each direction. Median read-depth, based on read length and number of reads mapping to the reference genome, was 73, IQR 36-99. Mapped read depth was assessed using bedtools v2.16.1 and the genomecov option, used to analyse the bam files generated by through mapping (Figure S3). Each mapped MTBC complex isolate was examined for mutations known to confer a resistant phenotype (Table S2). A minimum sequencing depth of five base calls was required for phenotype to be predicted based on a specific resistance-conferring variant being identified; where minority variant mutations were present with a depth of ≥5 and comprising ≥10% of the total base calls no single base was called and a mixed phenotype was predicted.

Genomic matches were identified using a rapid nearest neighbour finding algorithm. A maximum likelihood tree (R v3·1·2 with ape v3·2) was created from a set of 2191 previously sequenced isolates and the single nucleotide polymorphism (SNP) differences between adjacent nodes of the tree were stored in a database. Previously sequenced isolates were collected and sequenced as part of separate investigations from the Regional Mycobacterial Reference Laboratory, Birmingham, between 1996 and 2012 and Oxford University Hospitals between 2007 and 2012.[1-3]Also included were previously published sequences from Gardy *et al* (2011) with a minority of sequences obtained from other available European samples processed between 2011-2013.[4] Newly sequenced isolates could then be queried against the database in real-time, with the algorithm reporting all matches within 20 SNPs of the queried isolate or the single closest match if all differences were >20 SNPs. Isolates within or equal to five SNPs were considered compatible with recent direct or indirect transmission, given within host evolution and observed genetic differences within known household outbreaks, with isolates 6-12 SNPs distant possibly compatible with transmission.[1] The SNP differences between each queried isolate and the adjacent node were stored in a 'bucket' at the node, allowing new isolates to be iteratively added to the database. This system avoided the high computational cost of tree reconstruction with the addition of each new isolate. Subclades of the

tree would need to be recalculated when buckets became overpopulated, but because the number of isolates included in this study was relatively small, no subclade or tree reconstruction was required during the course of this investigation. (Identifying when a subclade or tree would need to be reconstructed is an important question for future work.) All isolates reported as nearest neighbours were compared to the queried isolate by pairwise alignment and maximum-likelihood trees (PhyML v3·0) constructed from concatenated variable sites.

Analysis

Following the full WGS report being issued, data from routine and reference laboratory processing of the same isolate were gathered by local laboratory staff and returned to Public Health England (PHE) Oxford for analysis. All data were fully anonymised.

Data collected from routine and reference laboratories included: the date of sample collection from the patient, or when the sample was received by the routine laboratory; whether the sample was a duplicate (from the same patient) of a previously processed sample; the date that the MGIT became positive; the date that an aliquot of the MGIT was sent to the reference laboratory; the date on which species information was obtained from the reference and/or local laboratory; reference and/or local laboratory species result for the isolate; and for isolates identified as MTBC by the routine laboratory, the date on which drug sensitivity profiles were obtained from the reference laboratory; the drugs tested by the reference laboratory; the drug sensitivity profile of the isolate; the date on which MIRU-VNTR data were obtained from the reference laboratory; and the MIRU-VNTR profile of the isolate.

Data collected throughout WGS processing and analysis included: the date of sample extraction; the date WGS was performed; the date WGS data were shared via BaseSpace; the date of species identification; the date of drug sensitivity prediction; the date of nearest neighbour matching; and the date a full WGS report was returned to the participating centre.

Data were compiled at the John Radcliffe Hospital, Oxford, UK, and analysed in Stata 13·1 (StataCorp, USA). Routine species identification and drug sensitivity profiles were treated as the reference standard for comparison to WGS results; discrepancies were re-tested using routine methods where possible but isolates were not re-sequenced. As specimens were anonymised, primary analysis including comparisons with species and resistance results from routine laboratories was performed for all specimens. Identification of duplicate MTBC specimens was performed by each participating centre, and data regarding the duplication of non-MTBC specimens were not collected. MTBC de-duplication was performed by selecting the first sample taken from each patient (where multiple samples taken from the same patient), or by selecting the second sample where WGS had been re-performed due to sequencing technical failure. Both the full dataset (all specimens) and de-duplicated data set were utilised for outbreak analysis and statistical analysis of drug-resistance. The time for each stage of the diagnostic workflow to complete was compared across routine and WGS processing, excluding isolates where routine processing was not performed in full (for example where, clinical diagnosis was performed based on previously processed isolates from the same patient, as identified by participating centres). Multivariable fractional polynomial logistic regression was performed to identify which quality control measures contributed to WGS sequence processing failure (Stata mfp; exit p=0·05).

Costs

A micro-costing questionnaire based on standard operating procedures and diagnostic algorithms was completed by a regional reference laboratory (the Regional Centre for Mycobacteriology, Birmingham, UK), and a local clinical laboratory performing WGS (Oxford, UK). The local laboratory did not perform the full routine mycobacterial workflow. Other participating centres in the UK declined to participate. Questionnaires were completed by clinical scientists and financial managers. Accuracy of the questionnaire, clinical and WGS workflows, and the associated costs were ensured through expert consultations and interview with clinical scientists performing the tests, and financial managers. The questionnaire gathered bottom-up costs for the full procedure from the clinical sample being received by the routine laboratory to data interpretation and reporting; this included consumables, hardware (computing and laboratory equipment; initial cost, maintenance and proportion of time used for MTBC diagnostics), staff time, staff training time, annual staff turnover, equipment calibration, service contracts, and reported error rates (Table S3). For second-line drug phenotyping, only costs based on staff time, consumables and equipment were gathered from the National Mycobacterial Reference Laboratory, London, UK, via interview with clinical scientists. Throughput, used to annualise costs of both diagnostic workflows, was based on reported sample numbers in Birmingham for 2014.

Routine processing of clinical samples was based on procedures at Birmingham, and included sample receipt, MGIT culture, Cepheid Xpert MTB/RIF assay, species identification for MGIT cultures using either the Hain GenoType MTBC or Hain GenoType Mycobacterium CM/AS assays, MIRU-VNTR, phenotyping for first-line drug susceptibility testing (DST) (MGIT culture), Hain MTBDR*plus* for first-line DST, secondary phenotyping for first-line DST (Lowenstein-Jensen (LJ) media), Hain MTBDR*sl* for second line DST and sending off drug-resistant isolates for full second-line phenotyping at an external laboratory.

Staff salaries published by the NHS Agenda for Change were taken from the year 2014. Hardware costs were attached to equipment from laboratory price listings (Table S3). As per guidance of the National Institute for Health and Care Excellence, cost figures do not include VAT. For capital items the cost was spread over the item's predicted lifetime and depreciated using equivalent annual costing with a discount rate of 3·5%. Of the total costs, a 20% rate was added for overheads including items such as general hospital administration, cleaning and electricity. In addition, a rate of 20% for national insurance and superannuation was included in the analysis and it was assumed that staff work 37·5 hours per week and 46 weeks per year.

A sensitivity analysis was performed to assess how pricing changes would affect the cost of overheads, WGS equipment and WGS major consumables. Alterations in the annual throughput of specimens and the specimen batch size of WGS were included, alongside changes in the staff grade performing laboratory work, error rates, and the lifetime of routine and WGS laboratory equipment (Tables S9 and S10).

Figure S1: Flow diagram of suspected Mycobacterium sample through the current diagnostic pathway and the WGS diagnostic pathway used during this investigation. Timings for current diagnostic pathway are typical for routine and Public Health England (PHE) reference laboratories in the UK and may vary. Ziehl-Neelsen (ZN) positive samples typically become BACTEC Mycobacterial Growth Indicator Tube (MGIT) positive 7-10 days from inoculation. ZN negative samples can become BACTEC MGIT positive up to 35 days from inoculation. See Figure S2 for detail of data analysis. Green shading = routine and reference laboratory processing; blue shading = WGS processing; purple shading = reporting.

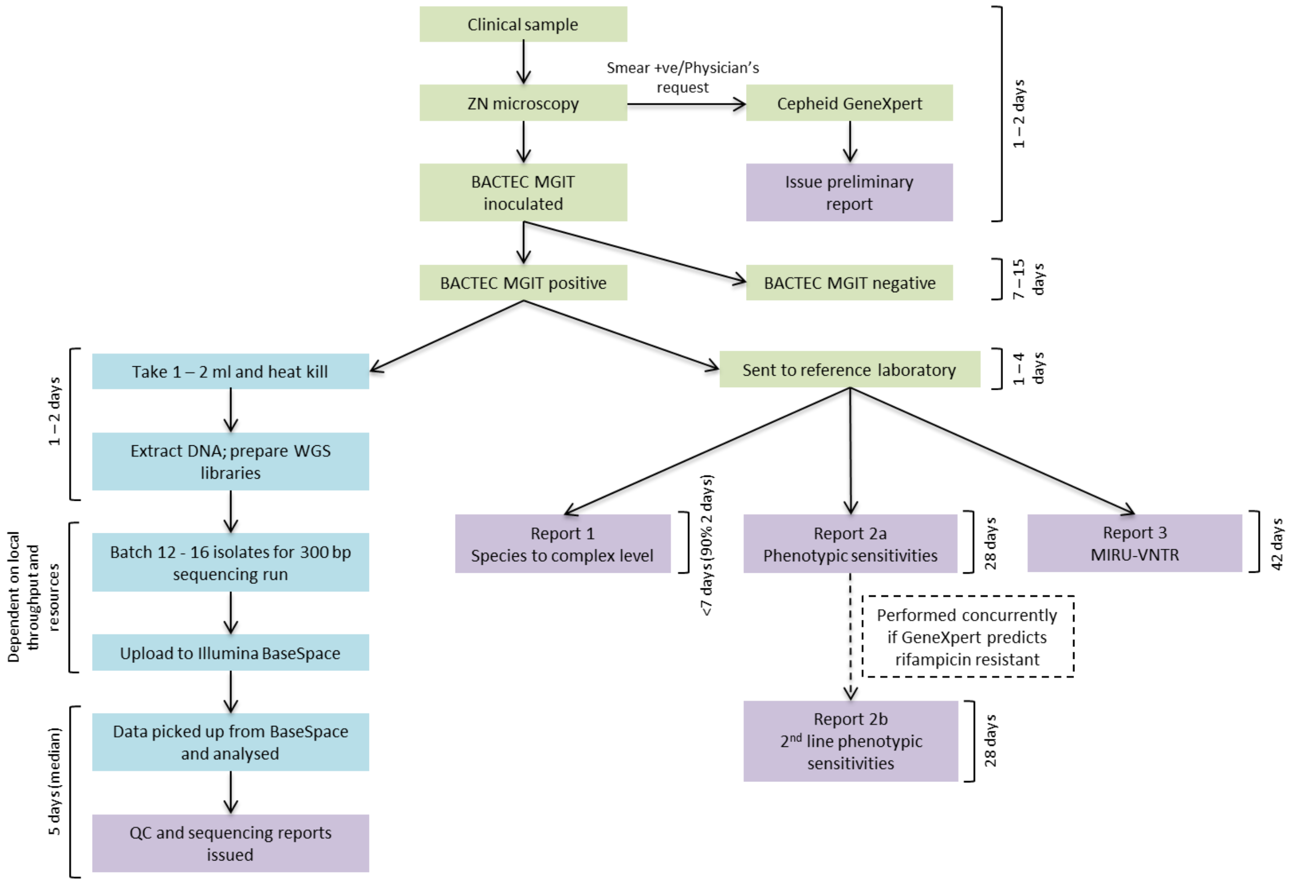

Figure S2: Flow diagram of WGS data through processing pipeline. Blue shading = WGS processing; purple shading = reporting; no shading = manual processing steps. QC = Quality control.

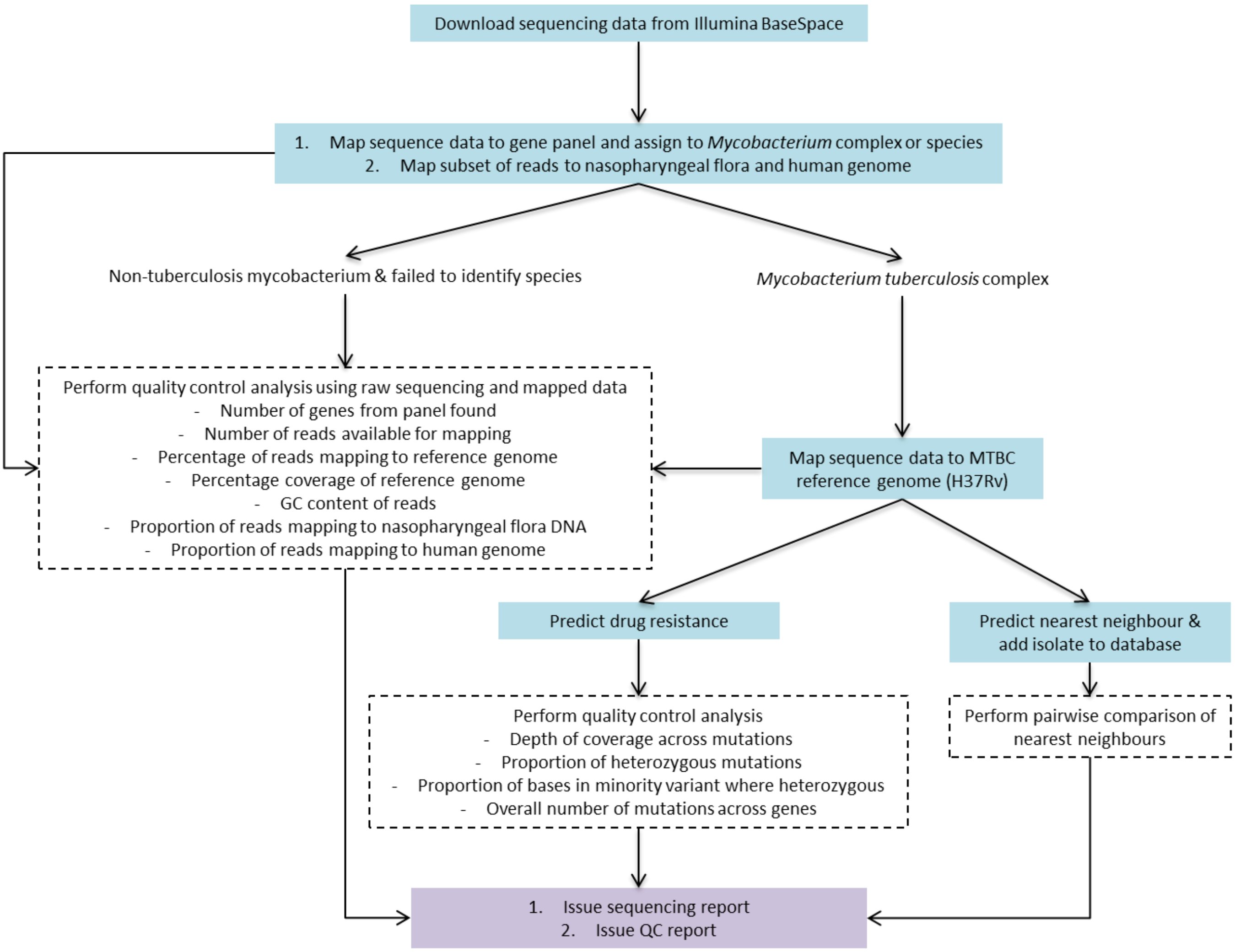

Figure S3: Histogram of the mapped read depth of MTBC samples found during this investigation.

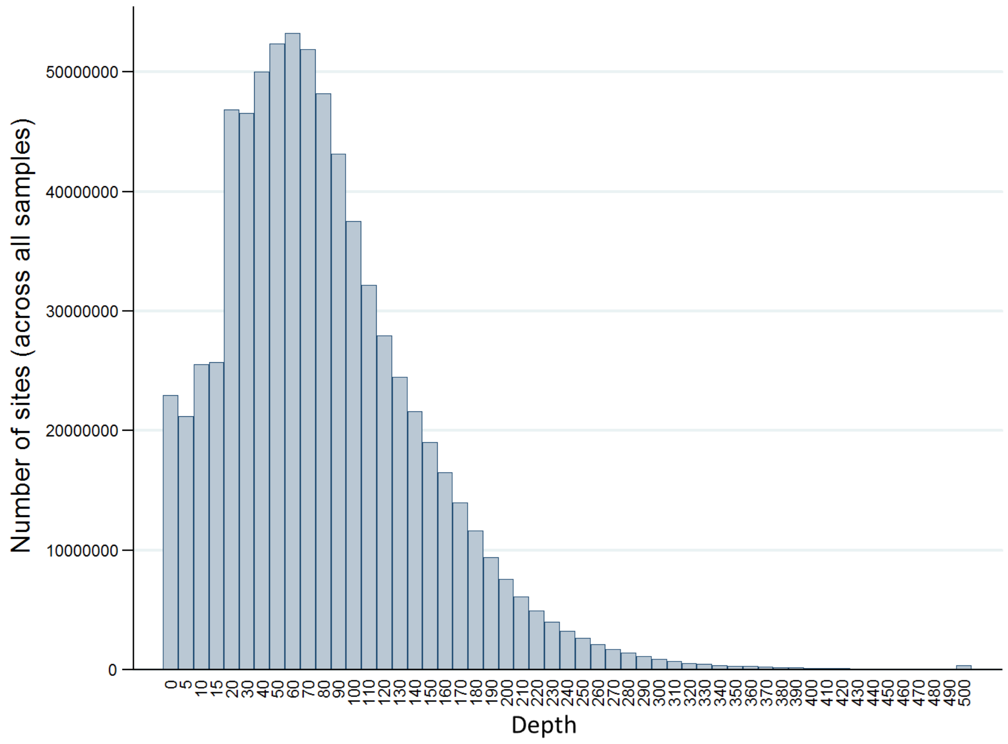

Note: One observation per reference genome site per mapped sample.

Figure S4: Example of prototype quality checking report completed and returned to sequencing centres for each isolate.

QC Report

Sample Details			
Sequencing Location	Oxford	Date Received in Lab	4th Nov 2013
Local LIMS Specimen ID	M5979	Run Date	6th Dec 2013
GUID	N/A		

Sequencing run Statistics	
GC Content	65%
Median Insert Size	302
Reads For Mapping	4091106
Reads Mapped to TB Reference	3995924
Percentage Mapped to TB Reference	97.7%
Coverage of TB Reference	91.7%
Percentage Mapped to Human Genome	0%
QC comment	

Figure S5: Example of prototype sequencing report completed and returned to sequencing centres for each isolate. GUID = Global Unique Identifier; 'Ambiguous' resistotype = mixed phenotype prediction.

Whole Genome Sequencing Report from MGIT Positive Samples

Not for diagnostic use

Sample Details			
Sequencing Location	Oxford	Date Received in Lab	4th Nov 2013
Local LIMS Specimen ID	M5979	Run Date	6th Dec 2013
GUID	N/A		

Sample/Sequencing Quality	
Comments	

Organism Identification
Mycobacterium tuberculosis

Resistotype				
Drug	Prediction	HAIN Mutation	Extended Catalogue	Ambiguous
Isoniazid	Sensitive			
Rifampicin	Sensitive			
Ethambutol	Sensitive			
Pyrazinamide	Sensitive			
Streptomycin	Sensitive			
Moxifloxacin	Sensitive			
Amikacin	Sensitive			

Relatedness			
Nearest neighbour(s)			Genealogy
GUID	No. of SNPs Apart	Centre	
C00016533	2	Oxford	
C00023214 (M6076)	0	Oxford	
C00023211 (M5992)	0	Oxford	

Authorised	
Signature:	Print name: Timothy Walker
Position:	Date: 12th December 2013

Figure S6: Total number of sequencing reads generated per sample; samples categorised according to WGS result using routine laboratory results as reference standard. Successful WGS species identification defined as complete concordance, loss/gain of NTM in co-infection, identification of subspecies. Failed WGS species identification defined as no species identified, loss of MTBC and discordance. The total number or reads were truncated at 7×10^6. Inverse association between total number of reads and failure (power -0·5); p=0·005. *WGS & routine lab failed: WGS failed owing to low read numbers and/or high levels of contamination with non-mycobacterial DNA; no mycobacterial genes found by species presence/absence algorithm.

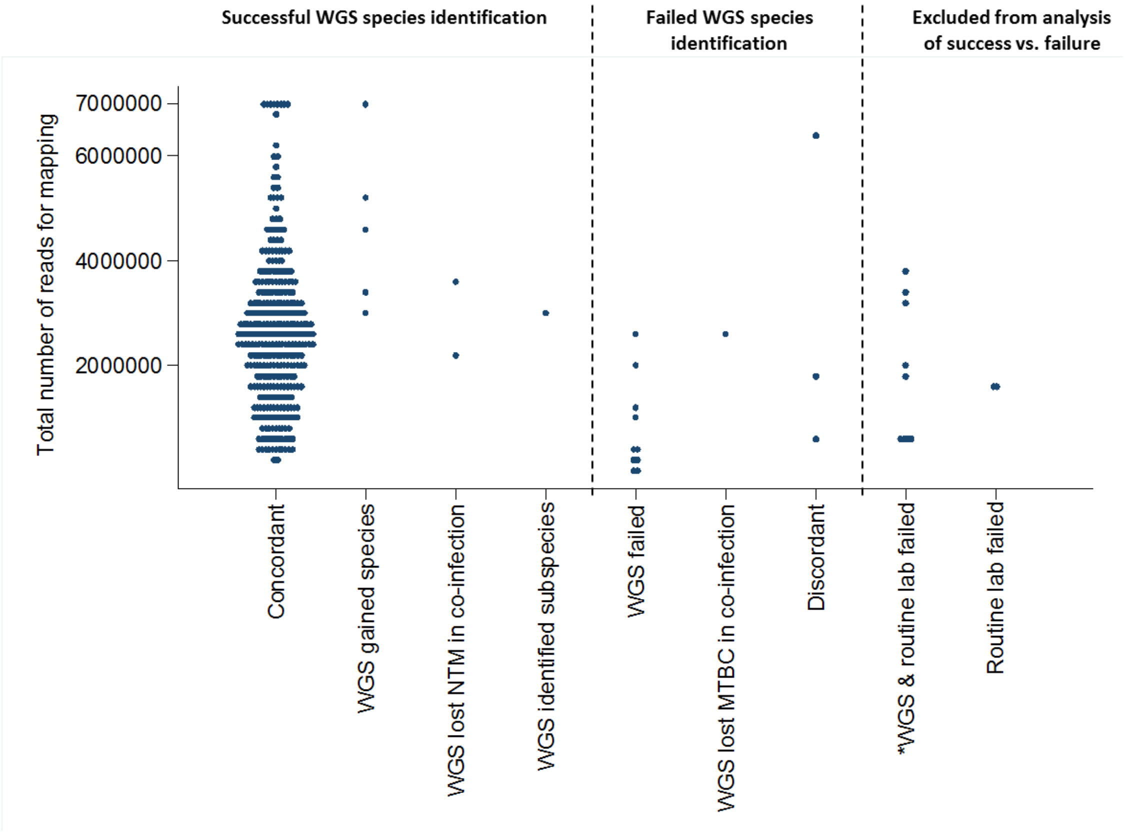

Figure S7: The percentage GC content of each sample; samples categorised according to WGS result using routine laboratory results as reference standard. Successful WGS species identification defined as complete concordance, loss/gain of NTM in co-infection, identification of subspecies. Failed WGS species identification defined as no species identified, loss of MTBC and discordance. Inverse association between GC content and failure (power 1); p<0·001. *WGS & routine lab failed: WGS failed owing to low read numbers and/or high levels of contamination with non-mycobacterial DNA; no mycobacterial genes found by species presence/absence algorithm.

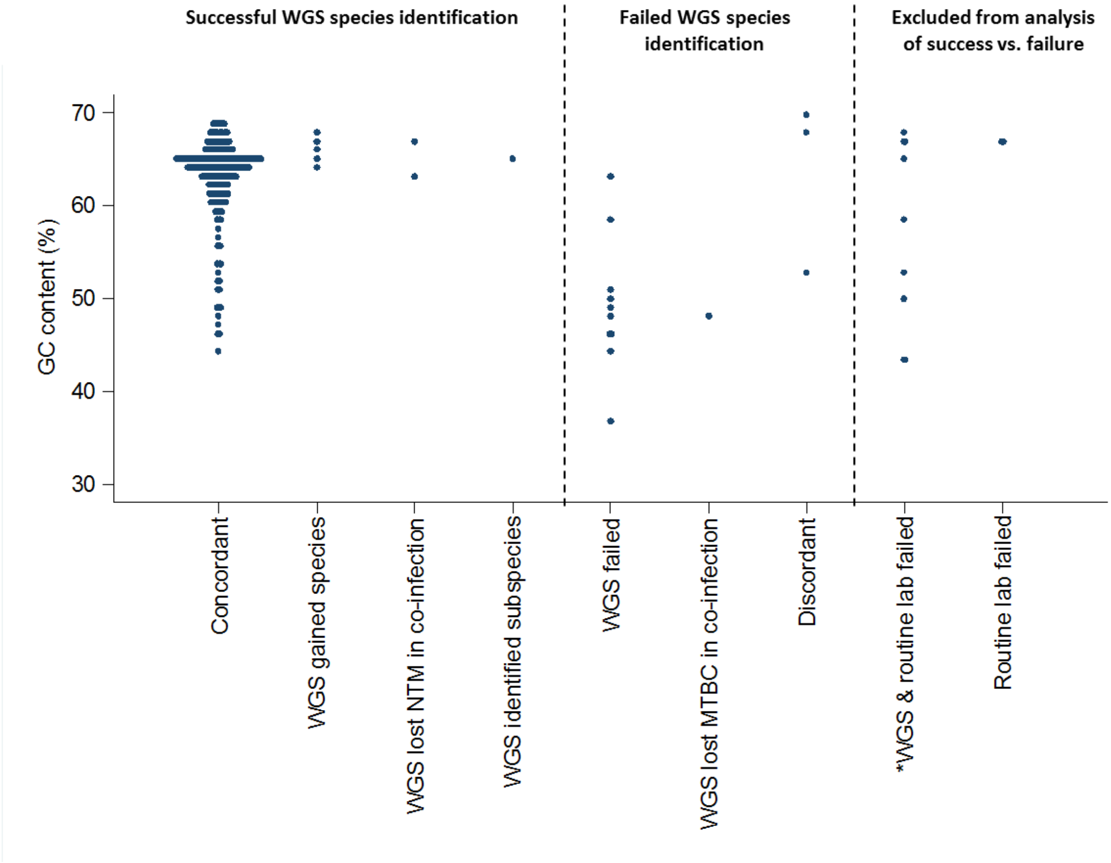

Figure S8: The predicted probability of WGS failing to identify or incorrectly identifying species depended independently on the number or reads available for mapping (p=0.005) and GC content (p<0.001) based on a multivariable fractional polynomial logistic regression model .[5] This allows the effect of each predictor to vary non-linearly, i.e. for each unit increase in the predictor to have a different impact on the odds of success across the range of values taken by the predictor. Area under the receiver-operating curve =0.90.

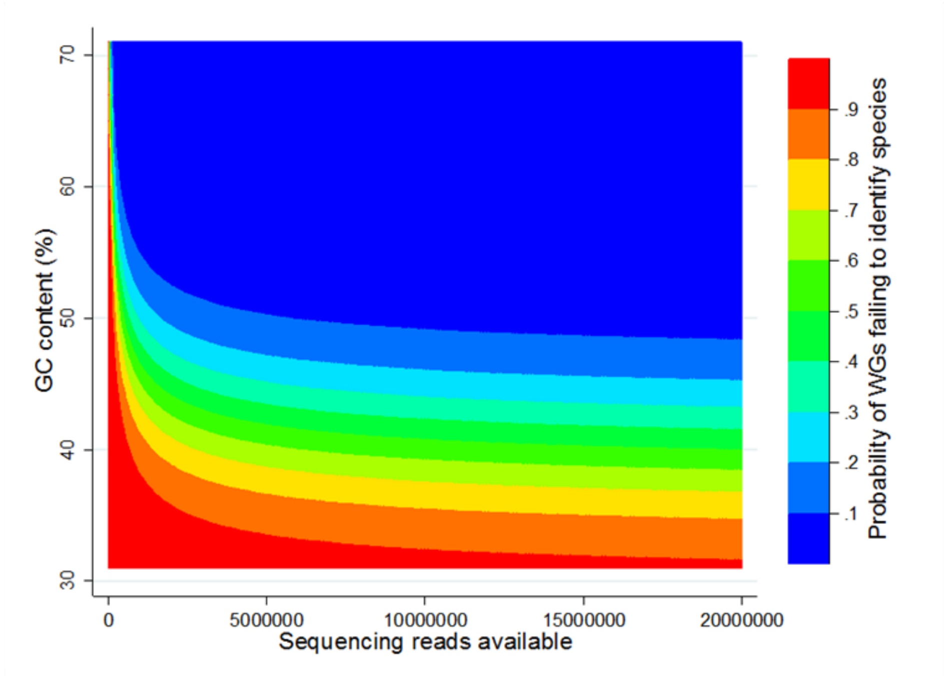

Figure S9: Workflow at Regional Centre for Mycobacteriology (Birmingham, UK) used for costing analysis with costs for each diagnostic step and cumulative costs of diagnosis by both routine methods, and WGS if WGS was to replace all routine diagnostic steps, or species identification and MIRU-VNTR only. *Performed at regional centre. ‡Performed at National Centre for Mycobacteriology (London, UK)

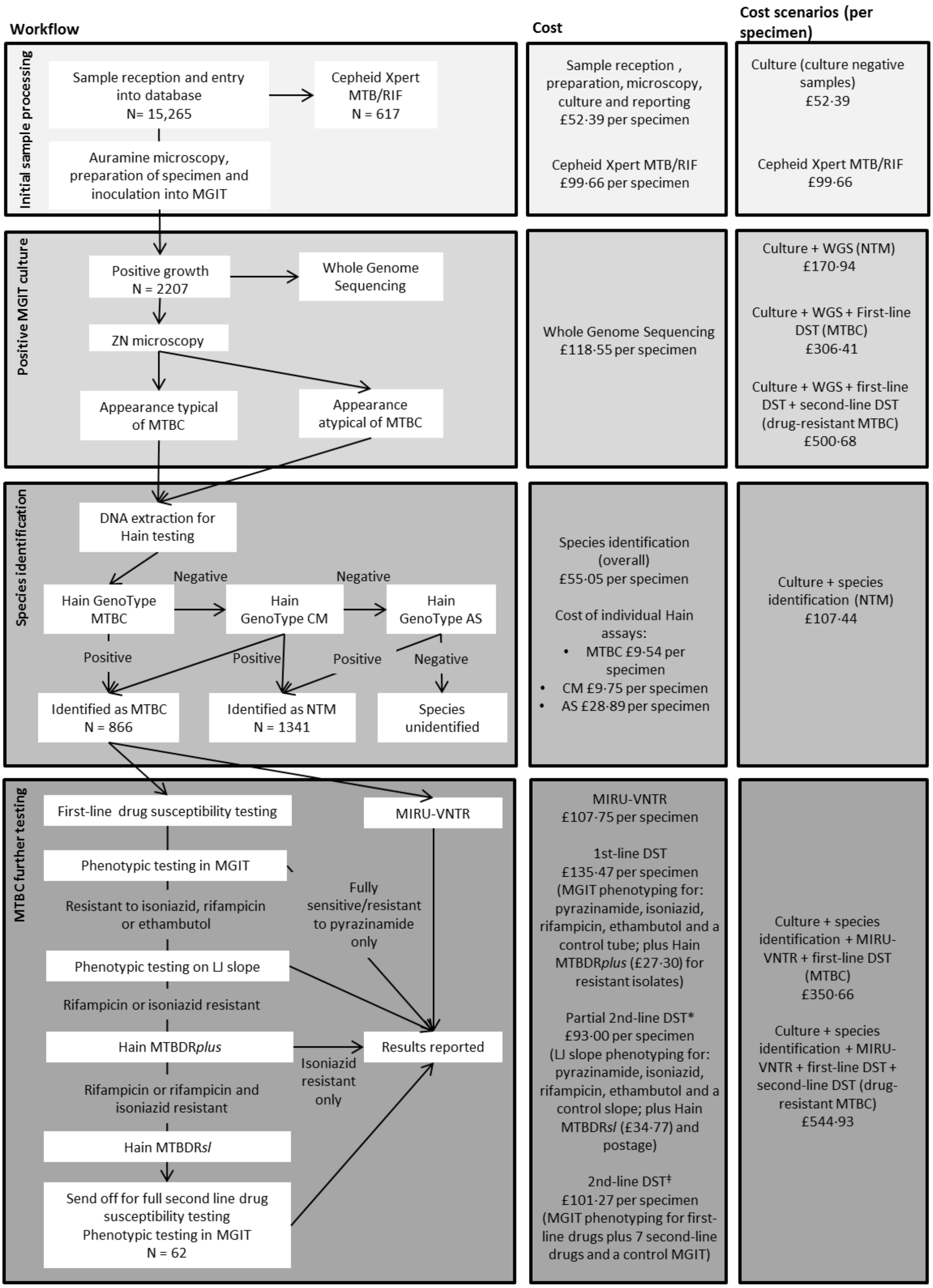

Figure S10: Percentage of total cost split by cost category

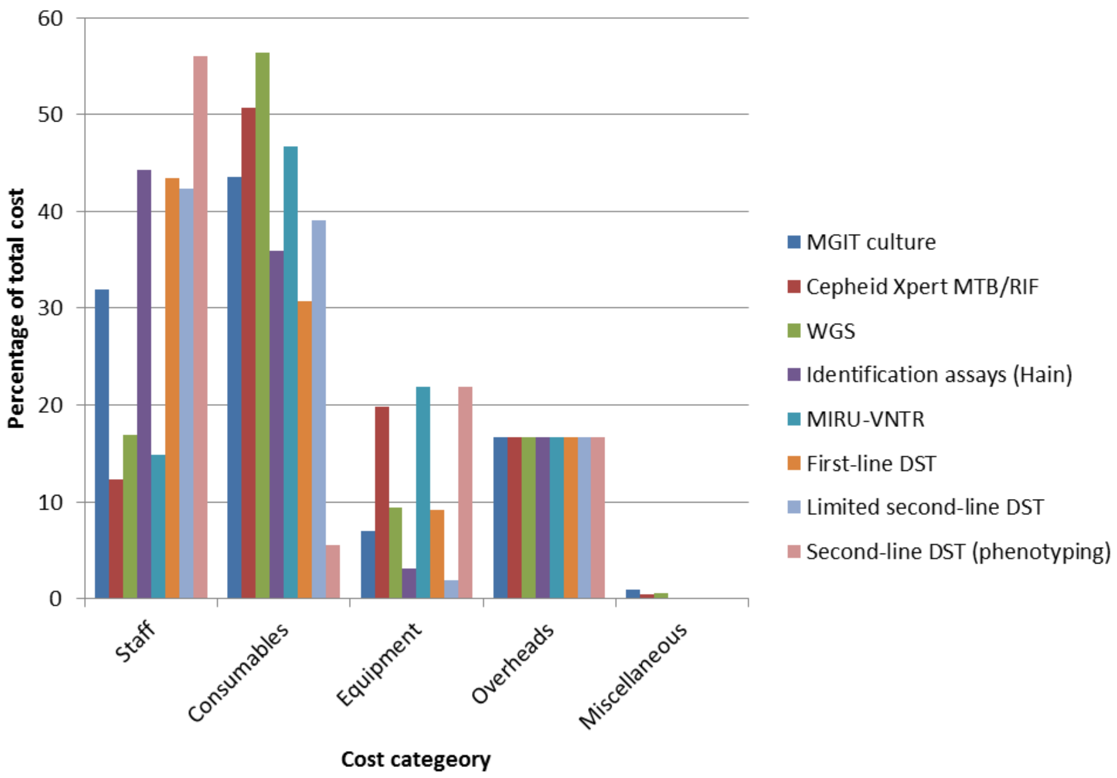

Table S1: Sample collection at each participating centre; dates of MGIT collection and summary of routine laboratory species identification results.

Participating centre	MGIT collection		Routine laboratory species identification (N)				
	**Start date**	**End date**	***Mycobacterium tuberculosis* complex**	**Non-tuberculous mycobacteria**	**Co-infection (MTBC+NTM)**	**Failed to identify**	**Total**
John Radcliffe Hospital, Oxford University Hospitals NHS Foundation Trust, Oxford (UK)	10th October 2013	22nd February 2014	33	59	1	5	98
Leeds Teaching Hospitals NHS Trusts (UK)	11th October 2013	25th January 2014	42	41	0	4	87
Brighton and Sussex University Hospitals NHS Trust (UK)	6th September 2013	8th January 2014	10	27	0	1	38
Birmingham Heartlands Hospital NHS Foundation Trust (UK)	31st January 2014	14th April 2014	32	19	0	1	52
St James' Hospital, Dublin (Ireland)	2nd October 2013	9th March 2014	13	9	1	0	23
Forschungs Institute, Borstel (Germany)	3rd February 2014	14th April 2014	34	0	0	0	34
University Hospital Lille (France)	25th December 2013	28th February 2014	6	6	1	0	13
British Columbia Centre for Disease Control, Vancouver (Canada)	27th November 2013	6th December 2013	5	5	1	0	11

Table S2: Catalogue of mutations examined for phenotypic resistance prediction. Mutations selected from Hain line probe assays and in-house catalogues based on literature searches, as previously described.[6]

Drug	Catalogue	Mutation
Isoniazid	Hain	fabG1_*-15*
		fabG1_*-16*
		fabG1_*-8*
		katG_*315*
	Extended catalogue	ahpC_C-39T
		ahpC_G-46A
		katG_G279D
		katG_T180K
		katG_T302R
		katG_V473F
		katG_Y300C
Rifampicin	Hain	rpoB_*425*
		rpoB_*426*
		rpoB_*427*
		rpoB_*428*
		rpoB_*429*
		rpoB_*430*
		rpoB_*431*
		rpoB_*432*
		rpoB_*433*
		rpoB_*434*
		rpoB_*435*
		rpoB_*436*
		rpoB_*437*
		rpoB_*438*
		rpoB_*439*
		rpoB_*440*
		rpoB_*441*
		rpoB_*442*
		rpoB_*443*
		rpoB_*444*
		rpoB_*445*
		rpoB_*446*
		rpoB_*447*
		rpoB_*448*
		rpoB_*449*
		rpoB_*450*
		rpoB_*451*
		rpoB_*452*
Ethambutol	Hain	embB_*306*
	Extended catalogue	embB_G406D

		embB_G406S
		embB_Q1002R
		embB_Q497R
		embB_T506N
		embB_W332R
Pyrazinamide	Extended catalogue	pncA_A-11G
		pncA_A102V
		pncA_A134V
		pncA_A146E
		pncA_A161P
		pncA_A171E
		pncA_A46V
		pncA_C138R
		pncA_C138Y
		pncA_C14R
		pncA_C72R
		pncA_D12A
		pncA_D63G
		pncA_D8G
		pncA_G162D
		pncA_G17D
		pncA_G78C
		pncA_G97D
		pncA_G97S
		pncA_H137R
		pncA_H51P
		pncA_H57D
		pncA_H57Y
		pncA_H71R
		pncA_H82R
		pncA_K96N
		pncA_K96T
		pncA_L116R
		pncA_L159P
		pncA_L172E
		pncA_L172P
		pncA_L19P
		pncA_L27P
		pncA_L35R
		pncA_L4S
		pncA_L85P
		pncA_P54T
		pncA_Q10P
		pncA_Q141P
		pncA_R121P

		pncA_R140S
		pncA_S104R
		pncA_S185T
		pncA_S66P
		pncA_S67P
		pncA_T-12C
		pncA_T114P
		pncA_T142K
		pncA_T160P
		pncA_T76P
		pncA_V125G
		pncA_V128G
		pncA_V130G
		pncA_V139L
		pncA_V155G
		pncA_V21G
		pncA_W68C
		pncA_W68G
		pncA_W68R
		pncA_Y34S
Streptomycin	Extended catalogue	gid_A138V
		gid_A200E
		gid_A200E
		gid_G34A
		gid_G71R
		gid_H48N
		gid_L91P
		gid_S100F
		gid_V65G
		gid_V88A
		rpsL_K43R
		rpsL_K88Q
		rpsL_K88R
		rrs_A514C
Ciprofloxacin, Ofloxacin and Moxifloxacin (Fluroquinolones)	Hain	gyrA_*85*
		gyrA_*86*
		gyrA_*87*
		gyrA_*88*
		gyrA_*89*
		gyrA_*90*
		gyrA_*91*
		gyrA_*92*
		gyrA_*93*
		gyrA_*94*
		gyrA_*96*

		gyrA_*97*
	Extended catalogue	gyrA_A74S
		gyrA_P102H
Amikacin, Capreomycin, Kanamycin (Aminoglycosides)	Hain	rrs_*1401*
		rrs_*1402*
		rrs_*1484*
	Extended catalogue	eis_G-10A

Table S3: Input parameters for costing estimates. *Items over £10,000

**Equipment***			
**Item**	**Total cost (GBP)**	**Annual maintenance (GBP)**	**Lifetime (Years)**
MiSeq (Illumina, USA)	83,282	9750	10
Tapestation 2200 (Agilent, USA)	12,000	0	5
Fluorescent microscope	11,914	0	10
GeneXpert (Cepheid, USA)	44,734	6576	10
QIAgility (Qiagen, USA)	26,000	0	10
WAVE System (Transgenomic, USA)	31,000	6593	10
MGIT 960 (Beckton Dickinson, USA)	25,000	2974	10
BeeBlot (Bee Robotics, UK)	18,500	0	10

**Key variables**	
**Variable**	**Value**
National Insurance/Superannuation Multiplier	1·2
Weeks worked per year	46
Hours worked per week	37·5
Overheads (%)	20
Discount rate (%)	3·5
**Error rates**	**%**
Microscopy	1
MGIT culture	2
Cepheid Xpert MTB/RIF	10
Species identification (Hain ID)	0·05
DNA extraction for Whole Genome Sequencing	13
Whole Genome Sequencing	4
Whole Genome Sequencing data analysis	1·4
MIRU-VNTR	10
Drug susceptibility testing	0·05

Table S4: Summary of isolates with part-concordant species results and subsequent re-testing results

Initial routine identification	WGS identification	WGS: Unique genes available	WGS: Unique genes identified	Re-testing performed	Re-testing result	Summary
M. tuberculosis complex	*M. tuberculosis* complex & *M. avium* complex	100 & 33	99 & 29	No	N/A	Unable to re-test WGS isolate.
M. avium complex	*M. tuberculosis* complex & *M. avium* complex	100 & 33	4 & 33	Yes. Re-sequenced new library preparation	*M. avium* complex	Re-testing supports routine identification. Potential contamination during original library preparation.
M. avium complex	*M. tuberculosis* complex & *M. avium* complex	100 & 33	84 & 33	Yes. Hain testing of WGS DNA extraction	*M. tuberculosis* complex & *M. avium* complex	Re-testing supports WGS identification. Potential contamination during original sample extraction
M. avium complex	*M. tuberculosis* complex & *M. avium* complex	100 & 33	34 & 33	Yes. Re-sequenced new library preparation	*M. avium* complex	Re-testing supports routine identification. Potential contamination during original library preparation.
M. abscessus complex	*M. abscessus* complex & *M. avium* complex	40 & 33	40 & 32	Yes. Hain testing of WGS DNA extraction	*M. abscessus* complex	Re-testing supports routine identification. Potential contamination during original sample preparation.
M. tuberculosis complex & *M. avium* complex	*M. tuberculosis* complex	100	97	Yes. Hain testing of WGS DNA extraction	*M. tuberculosis* complex	Re-testing supports WGS identification. Co-infection was confirmed in patient; suggesting *M. avium* in extract used for WGS was below detection limits.
M. tuberculosis complex & *M. avium* complex	*M. avium* complex	33	2	No	N/A	Patient with confirmed co-infection; poor quality sequencing (88% human DNA)
M. tuberculosis complex & *M. avium* complex	*M. avium* complex	33	24	No	N/A	Unable to re-test WGS isolate.
Undescribed mycobacterial species	*M. avium* complex	33	33	Yes (automated scanning of Hain and correspondence with Hain Lifescience)	Hain invalid	Initially identified as *M. avium* complex/*M. malmoense* co-infection, re-examination of the Hain test result and correspondence with Hain Lifescience led to identification of an undescribed mycobacterium bearing close relation to *M. avium*
M. fortuitum	*M. fortuitum-acetamidolyticum*	100	33	No	N/A	Poor quality sequencing, with only 33% *M. fortuitum-acetamidolyticum* genes identified by WGS.

Table S5: Summary of isolates with discordant species results and subsequent re-testing results

Routine identification	WGS identification	WGS: Unique genes available	WGS: Unique genes identified	Re-testing performed	Re-testing result	Summary
M. kansasii	*M. avium* complex	33	33	Yes. MGIT culture sent to reference laboratory for testing.	*M. avium* complex	Routine identification based on testing of previous isolate from this patient. Patient confirmed to be infected with *M. avium* complex after testing of same isolate used for WGS.
M. avium complex	*M. scrofulaceum*	72	1	No	N/A	Poor quality sequencing data (500,000 reads available for analysis)
M. tuberculosis complex	*M. abscessus* complex	40	38	No	N/A	Unable to re-test WGS isolate.

Table S6: Drug-resistance predicted by WGS and reference laboratory DST by MTBC specimen (N=168). S = Sensitive; R = Resistant; F = Failed prediction; M = Mixed reads with both wild-type and resistance-conferring mutation; . = Not tested.

Sample ID	Reference laboratory DST							WGS						
	**Isoniazid**	**Rifampicin**	**Ethambutol**	**Pyrazinamide**	**Streptomycin**	**Fluoroquinolones**	**Aminoglycosides**	**Isoniazid**	**Rifampicin**	**Ethambutol**	**Pyrazinamide**	**Streptomycin**	**Fluoroquinolones**	**Aminoglycosides**
140602981	S	S	S	S	.	.	.	S	S	S	S	S	S	S
140602172	S	S	S	S	.	.	.	S	S	S	S	S	S	S
140602480	S	S	S	S	.	.	.	S	S	S	S	S	S	S
140602321	S	S	S	S	.	.	.	S	S	S	S	S	S	S
140602881	S	S	S	S	.	.	.	S	S	S	S	S	S	S
140602189	S	S	S	S	.	.	.	S	S	S	S	S	S	S
140602173	S	S	S	S	.	.	.	S	S	S	S	S	S	S
140602632	S	S	S	S	.	.	.	S	S	S	S	S	S	S
140602055	S	S	S	S	.	.	.	S	S	S	S	S	S	S
140601636	S	S	S	S	.	.	.	S	S	S	S	S	S	S
140602933	F	S	S	S	.	.	.	S	S	S	S	S	S	F
140602863	S	S	S	S	.	.	.	S	S	S	S	S	S	S
140603011	S	S	S	S	.	.	.	S	S	S	S	S	S	S
140600095	S	S	S	S	.	.	.	S	S	S	S	S	S	S
140603272	S	S	S	S	.	.	.	S	S	S	S	S	S	S
140603568	S	S	S	S	.	.	.	S	S	S	S	S	S	S
140602707	S	S	S	S	.	.	.	S	S	S	S	S	S	S
140603170	S	S	S	S	.	.	.	S	S	S	S	S	S	S
140603683	S	S	S	S	.	.	.	S	S	S	S	S	S	S
140603704	S	S	S	S	.	.	.	F	F	F	F	F	F	F
140603310	R	R	S	S	.	R	.	S	S	S	M	S	R	S
140603751	S	S	S	S	.	R	.	S	S	S	S	S	S	F
140604083	S	S	S	S	.	.	.	S	S	S	S	S	S	S
140603858	S	S	S	S	.	.	.	S	S	S	S	S	S	S
140602314	S	S	S	S	.	.	.	S	S	S	S	S	S	S
140603921	R	S	R	R	.	R	.	M	S	R	S	S	R	S
140604540	S	S	S	S	.	.	.	S	S	S	S	S	S	M
140604530	S	S	S	S	.	.	.	S	S	S	S	S	S	S
140602969	S	S	S	S	.	.	.	S	S	S	S	S	S	S
140603481	R	R	R	S	.	R	.	R	R	R	S	S	R	S
140600470	S	S	S	S	.	.	.	S	S	S	S	S	S	S
566-14	S	S	S	S	.	.	.	S	S	S	S	S	S	S
307-14	S	S	S	S	R	.	.	S	S	S	S	R	S	S
570-14	S	S	S	S	R	.	.	S	S	S	S	R	S	S
965-14	S	S	S	S	.	.	.	S	S	S	S	R	S	S
966-14	S	S	S	S	.	.	.	S	S	S	S	R	S	S
2222-14	S	S	S	S	.	.	.	S	S	S	S	S	S	S
2202-14	S	S	S	S	.	.	.	S	S	S	S	S	S	S
2238-14	S	S	S	S	.	.	.	S	S	S	S	S	S	S
1710-14	S	S	S	S	.	.	.	S	S	S	S	S	S	S
2385-14	S	S	S	S	.	.	.	S	S	S	S	S	S	S
2234-14	S	S	S	S	.	.	.	S	M	S	S	S	S	S
2223-14	S	S	S	S	.	.	.	S	S	S	S	S	S	S

1964-14	S	S	S	S	.	.	.	S	F	S	S	S	S	S
2602-14	S	S	S	S	.	.	.	S	S	S	S	S	S	S
2536-14	S	S	S	S	.	.	.	S	S	S	S	S	S	S
2525-14	R	S	S	S	.	S	S	R	S	S	S	S	S	S
2714-14	R	S	S	S	.	S	S	R	S	S	S	S	S	S
2740-14	S	S	S	S	.	.	.	S	S	S	S	S	S	S
2535-14	S	S	S	S	.	.	.	S	S	S	S	S	S	S
2741-14	S	S	S	S	.	.	.	S	S	S	S	S	S	S
2928-14	S	S	S	S	.	.	.	S	S	S	S	S	S	S
2985-14	S	S	S	S	.	.	.	S	S	S	S	S	S	S
2921-14	S	S	S	S	.	.	.	S	S	S	S	S	S	S
2880-14	S	S	S	S	.	.	.	S	F	S	S	S	S	S
2970-14	S	S	S	S	.	.	.	S	S	S	S	S	S	S
2984-14	S	S	S	S	.	.	.	S	S	S	S	S	S	S
2713-14	R	S	S	S	.	S	S	R	S	S	S	S	S	S
2929-14	S	S	S	S	.	.	.	S	S	S	S	S	S	S
3533-14	S	S	S	S	.	.	.	S	S	S	S	S	S	S
3230-14	S	S	S	S	.	.	.	S	S	S	S	S	S	S
3271-14	S	S	S	S	.	.	.	S	S	S	S	S	S	S
3231-14	S	S	S	S	.	.	.	S	S	S	S	S	S	S
13U178650	S	S	S	S	S	.	.	S	S	S	S	S	S	S
13U179862	S	S	S	S	S	.	.	S	S	S	S	S	S	S
13U175523	S	S	S	R	.	.	.	S	S	S	R	S	S	S
13U180137	R	S	S	S	.	.	.	R	S	S	S	S	S	S
13U187526	S	S	S	S	.	.	.	S	S	S	S	S	S	S
13U190202_E2	S	S	S	S	.	.	.	S	S	S	S	S	S	S
13U180134_E2	R	S	S	S	.	S	S	R	S	S	S	S	S	S
IMRL1	S	S	S	S	S	.	.	S	S	S	S	S	S	M
IMRL3	S	S	S	S	S	.	.	S	S	S	S	S	S	S
IMRL4	S	S	S	S	S	.	.	S	S	S	S	S	S	M
IMRL5	S	S	S	S	S	.	.	S	M	S	S	S	S	M
IMRL7	S	S	S	S	S	.	.	S	S	S	S	S	S	S
IMRL8	S	S	S	S	S	.	.	S	S	S	S	S	R	S
IMRL9	S	S	S	S	S	.	.	S	S	S	S	S	S	M
IMRL10	S	S	S	S	S	.	.	S	S	S	S	S	S	S
IMRL11	S	S	S	S	S	.	.	S	S	S	S	S	S	S
IMRL14	S	S	S	S	S	.	.	S	S	S	S	S	S	F
IMRL15	R	R	S	S	R	S	S	R	R	R	S	S	S	S
IMRL16	R	S	S	R	S	.	.	S	S	S	R	S	S	S
IMRL24	R	S	S	S	S	.	.	R	S	S	S	S	S	M
7626433	S	S	S	S	.	.	.	S	S	S	S	S	S	S
7626690	S	S	S	S	.	.	.	S	S	S	S	S	S	S
7626487	S	S	S	S	.	.	.	S	S	S	S	S	S	S
7625776	S	S	S	S	.	.	.	F	F	F	F	F	F	F
7626555	S	S	S	S	.	.	.	S	S	S	S	S	S	S
7626713	S	S	S	S	.	.	.	S	S	S	S	S	S	S
7626878	S	S	S	S	.	.	.	S	S	S	S	S	S	S
7626930	R	R	R	S	R	S	R	R	R	R	S	R	S	R
7626418.s2	S	S	S	S	.	.	.	S	S	S	S	S	S	S
7626586.s2	S	S	S	S	.	.	.	F	F	F	F	F	F	F
7626944.s2	S	S	S	S	.	.	.	F	F	F	F	F	F	F
7626833.s2	S	S	S	S	.	.	.	S	S	S	S	S	S	S
7626987.s2	S	S	S	S	.	.	.	F	F	F	F	F	F	F
7626571	S	S	S	S	.	.	.	S	S	S	S	S	S	S

7626675	R	R	R	S	R	.	.	R	R	R	S	R	S	R
7626694	S	S	S	S	.	.	.	S	S	S	S	S	S	S
7626725	S	S	S	S	.	.	.	S	S	S	S	S	S	S
7627124	S	S	S	S	.	.	.	S	S	S	S	S	S	S
7627311	S	S	S	S	.	.	.	S	S	S	S	S	S	S
7627400	S	S	S	S	.	.	.	S	S	S	S	S	S	S
7626752	S	S	S	S	.	.	.	S	S	S	S	S	S	F
7627116	S	S	S	S	.	.	.	S	S	S	S	S	S	S
7627226	R	R	R	S	R	.	.	R	R	R	S	R	S	R
7627572	S	S	S	S	.	.	.	S	S	S	S	S	S	S
7627574	S	S	S	S	.	.	.	S	S	S	S	S	S	S
7627636	S	S	S	S	.	.	.	S	S	S	S	S	S	S
7627434	S	S	S	S	.	.	.	S	S	S	S	S	S	S
7627736	S	S	S	S	.	.	.	S	S	S	S	S	S	S
7627886	S	S	S	S	.	.	.	S	S	S	S	S	S	S
7627900	S	S	S	S	.	.	.	S	S	S	S	S	S	S
7628147	S	S	S	S	.	.	.	S	M	S	S	S	S	S
7628143	S	S	S	S	.	.	.	S	S	S	S	S	S	S
7628121	S	S	S	S	.	.	.	S	S	S	S	S	S	S
7620009	S	S	S	S	.	.	.	S	S	S	S	S	S	S
7620018	S	S	S	S	.	.	.	S	S	S	S	S	S	S
7620179	S	S	S	S	.	.	.	F	F	F	F	F	F	F
7620215	R	S	S	S	.	.	.	R	S	S	S	S	S	S
7627873	S	S	S	S	.	.	.	F	F	F	F	F	F	F
7620149	S	S	S	S	.	.	.	S	S	S	S	S	R	S
7620040	S	S	S	S	.	.	.	S	S	S	S	S	S	S
7620062	S	S	S	S	.	.	.	S	S	S	S	S	S	S
Lil71	S	S	S	S	.	.	.	S	S	S	S	S	S	S
Lil73	S	S	S	S	.	.	.	S	S	S	S	S	S	S
Lil77	S	S	S	S	.	.	.	S	S	S	S	S	S	S
555710	F	F	F	F	.	.	.	F	F	F	F	F	F	F
Lil66	S	S	S	S	.	.	.	S	S	S	S	S	S	S
Lil68	S	S	S	S	.	.	.	S	S	S	S	S	S	S
W35519	S	S	S	S	.	.	.	S	S	S	S	S	S	S
H27078	R	S	S	S	.	.	.	R	S	S	S	S	S	S
M3785	S	S	S	S	.	.	.	S	S	S	S	S	S	S
L42182	S	S	S	S	.	.	.	S	S	S	S	S	S	S
L42183	S	S	S	S	.	.	.	S	S	S	S	S	S	S
L44277	S	S	S	S	.	.	.	S	S	S	S	S	S	S
F24817	S	S	S	S	.	.	.	S	S	S	S	S	S	S
H29889	S	S	S	S	.	.	.	S	S	S	S	S	S	S
M5992	S	S	S	S	.	.	.	S	S	S	S	S	S	S
F24816	S	S	S	S	.	.	.	S	S	S	S	S	S	S
L46496	S	S	S	S	.	.	.	S	S	S	S	S	S	S
M6076	S	S	S	S	.	.	.	S	S	S	S	S	S	S
L49781	S	S	S	R	.	.	.	S	S	S	R	S	S	S
M5979	S	S	S	S	.	.	.	S	S	S	S	S	S	S
L48766	S	S	S	S	.	.	.	S	S	S	S	S	S	S
W37690	S	S	R	R	.	.	.	S	S	S	R	S	S	S
H32592.I2	S	S	S	S	.	.	.	S	S	S	S	S	S	S
W41861.I2	S	S	S	S	.	.	.	S	S	S	S	S	S	S
L11705.I2	S	S	S	S	.	.	.	S	S	S	S	S	S	S
T62839	S	S	S	R	.	.	.	S	S	S	R	S	S	S
W41274	S	S	S	S	.	.	.	S	S	S	S	S	S	S

W41858	S	S	S	S	.	.	.	S	S	S	S	S	S	S
W41856	S	S	S	S	.	.	.	S	S	S	F	S	S	S
W41856_I2	S	S	S	S	.	.	.	S	S	S	S	S	S	S
W41860	S	S	S	S	.	.	.	S	S	S	S	S	S	S
W41858_I2	S	S	S	S	.	.	.	S	S	S	S	S	S	S
F28999	S	S	S	S	.	.	.	S	S	S	S	S	S	S
H34227	S	S	S	R	.	.	.	S	S	S	R	S	S	S
L17046	S	S	S	R	.	.	.	S	S	S	R	S	S	S
L17643	S	S	S	R	.	.	.	S	S	S	R	S	S	S
T64606	S	S	S	S	.	.	.	S	S	S	S	S	S	S
W44412.I2	S	S	S	S	.	.	.	S	S	S	S	S	S	S
W44411	S	S	S	S	.	.	.	S	S	S	S	S	S	S
H4354_s2	S	S	S	S	.	.	.	S	S	S	S	S	S	S
H4167_s2	S	S	S	S	.	.	.	S	M	S	S	S	S	S
T3020_s2	S	S	S	S	.	.	.	S	S	S	S	S	S	M
W4037_s2	S	S	S	S	.	.	.	S	S	S	S	S	S	S
W1592_s2	S	S	S	S	.	.	.	S	S	S	S	S	S	S

Table S7: Summary of the eight occasions across six patients with discordant sensitivity results and subsequent screening for additional mutations

Drug	Sample ID	DST (Phenotype)	WGS prediction	Candidate mutation	Summary
Isoniazid	140603310	Resistant	Sensitive	katG_W328L	Candidate mutation reported in resistant isolates in another study[7]
	IMRL16	Resistant	Sensitive	None	BCG isolate where low-level intrinsic resistance is reported[8]
Rifampicin	140603310	Resistant	Sensitive	rpoB_V170F	Candidate mutation reported in resistant isolates in another study[9]
Ethambutol	W37690	Resistant	Sensitive	embB_G406A	Hain testing confirmed no Hain mutation conferring resistance was present. Candidate mutation reported in resistant isolates in another study[10]
	IMRL15	Sensitive	Resistant	embB_M306V	Candidate mutation known to confer resistance but has previously been associated with sensitive phenotypes[11]
Pyrazinamide	140603921	Resistant	Sensitive	pncA_V7L	No support in available literature for this mutation. Other mutations at this codon have previously been reported in resistant isolates[12-14]
Streptomycin	IMRL15	Resistant	Sensitive	gidB_S100F	Mutation seen in both sensitive and resistant isolates[15]
Fluroquinolones	140603751	Resistant	Sensitive	gyrB_S313R<br>gyrA_E21Q	gyrB_S313R not reported as resistance-conferring in available literature. gyrA_E21Q reported in sensitive isolates[16,17]

Table S8: Outbreak clusters identified during this investigation (UK MTBC only).

Pilot cluster ID	Known to HPU	Published cluster ID	Description	Number of previous database isolates		During this pilot study		
				≤5 SNPs from pilot isolate	6 – 12 SNPs from pilot isolate	Number of isolates sequenced	From individuals (N)	From contributing centres (N)
1	Yes	Cluster 7[1]	Substance misuse[1]	8	19	1	1	1
2	Yes	Cluster 4[1]	School[1]	1	63	2	2	1
3	Yes	Cluster 3[1]	School[1]	7	0	1	1	1
4	Yes	Cluster 9[1]	Substance misuse[1]	21	9	1	1	1
5	Yes		Lifestyle	7	1	1	1	1
6	Yes		Lifestyle (interregional, isoniazid-resistant)	7	1	3	3	2
7	No		European MDR-TB	2	1	3	2	1
8	Yes	G5/E3[2]	Lifestyle	11	0	4	2	1
9	Yes		Social group	8	0	4	2	1

Note: Available MIRU-VNTR in pilot clusters 6, 7 and 9 were compatible with the genomic clusters

Table S9: Total cost per sample by process accounting for error rates. Error rates; 1% microscopy, 2% MGIT culture, 10% Cepheid Xpert MTB/RIF, 0·05% species identification (Hain ID), 13% DNA extraction for WGS, 4% WGS, 1·4% WGS data analysis, 10% MIRU-VNTR, DST 0·05%.

	Process	Costs by resource category (GBP)					Total per sample (GBP)
		Staff	Consumables	Equipment	Overheads	Miscellaneous*	
**WGS and routine clinical workflows**	MGIT culture	18·83	21·04	3·34	8·73	0·45	52·39
	Cepheid Xpert MTB/RIF	12·32	50·49	19·79	16·61	0·45	99·66
**WGS workflow only**	WGS	20·02	66·84	11·20	19·76	0·73	118·55
**Routine clinical workflows only**	Identification assays<br>*Hain MTBC*<br>*Hain CM/AS*	24·38	19·78	1·71	9·18	0·00	55·05
	MIRU-VNTR	15·95	50·32	23·52	17·96	0·00	107·75
	First-line DST	58·91	41·62	12·36	22·58	0·00	135·47
	Limited second-line DST‡	39·42	36·37	1·71	15·50	0·00	93·01
	Second-line DST‡‡	56·67	5·53	22·19	16·88	0·00	101·27

*For routine processing includes: annual staff turnover (10%), anemometer calibration and maintenance contract for category 3 laboratory. For WGS includes: annual staff turnover (10%) and staff training.

‡Performed at the Birmingham clinical laboratory

‡‡Performed at a second reference laboratory (London, UK). Based on staff time, consumables and equipment only.

Table S10: Staff time required, per specimen, for each workflow process and associated costs

	Grade 3	Grade 6	Grade 7	Grade 8	Total
Cost per hour (GBP)[18]	12·36	20·98	24·81	42·04	N/A
MGIT Culture					
Hands-on time (minutes)	6·13	35·44	11·99	N/A	53·56
Cost of error (GBP)	0·02	0·18	0·02	N/A	0·22
Total cost per sample (GBP)	1·28	12·57	4·98	N/A	18·83
Cepheid Xpert MTB/RIF					
Hands-on time (minutes)	1·54	18·90	11·99	N/A	32·43
Cost of error (GBP)	0·00	0·41	0·02	N/A	0·43
Total cost per sample (GBP)	0·32	7·02	4·98	N/A	12·32
Whole Genome Sequencing					
Hands-on time (minutes)	N/A	24·67	22·85	2.00	49·52
Cost of error (GBP)	N/A	0·50	0·05	0	0·55
Total cost per sample (GBP)	N/A	9·12	9·50	1.40	20·02
Hain GenoType MTBC/CM/AS					
Hands-on time (minutes)	N/A	95·92	3·00	N/A	68·92
Cost of error (GBP)	N/A	0·07	0·02	N/A	0·09
Total cost per sample (GBP)	N/A	23·12	1·26	N/A	24·38
MIRU-VNTR					
Hands-on time (minutes)	34·50	18·30	3·00	N/A	55·80
Cost of error (GBP)	0·71	0·47	0·02	N/A	1·20
Total cost per sample (GBP)	7·82	6·87	1·26	N/A	15·95
First-line DST					
Hands-on time (minutes)	N/A	164·61	3·00	N/A	167·61
Cost of error (GBP)	N/A	0·10	0·02	N/A	0·12
Total cost per sample (GBP)	N/A	57·65	1·26	N/A	58·91
Limited second-line DST					
Hands-on time (minutes)	N/A	108·92	3·00	N/A	111·92
Cost of error (GBP)	N/A	0·08	0·02	N/A	0·10
Total cost per sample (GBP)	N/A	38·16	1·26	N/A	39·42
Second-line DST (phenotyping)					
Hands-on time (minutes)	N/A	N/A	137·00	N/A	137·00
Cost of error (GBP)	N/A	N/A	0·03	N/A	0·03
Total cost per sample (GBP)	N/A	N/A	56·67	N/A	56·67

Table S11: Cost sensitivity analysis for routine clinical and WGS workflows. Shaded rows are current costs.

Variable	Adjustment value	MGIT Culture (GBP)	Cost change (%)	Cepheid Xpert MTB/RIF (GBP)	Cost change (%)	Whole Genome Sequencing (GBP)	Cost change (%)	Hain GenoType MTBC/CM/AS (GBP)	Cost change (%)	MIRU-VNTR (GBP)	Cost change (%)	First-line DST (GBP)	Cost change (%)	Limited second-line DST (GBP)	Cost change (%)	Second-line DST (phenotyping) (GBP)	Cost change (%)
Overheads	10%	48·02	-8·33	91·34	-8·33	108·68	-8·33	50·47	-8·33	98·77	-8·33	124·19	-8·33	85·26	-8·33	92·84	-8·33
	20%	52·39	0	99·66	0	118·55	0	55·05	0	107·75	0	135·47	0	93·01	0	101·27	0
	30%	56·75	8·33	107·95	8·33	128·44	8·33	59·64	8·33	116·73	8·33	146·76	8·33	100·77	8·33	109·72	8·33
Annual throughput	90%	52·90	0·97	102·35	2·71	120·16	1·34	55·28	0·41	110·89	2·91	137·12	1·22	93·24	0·25	104·24	2·92
	100%	52·39	0	99·66	0	118·56	0	55·05	0	107·75	0	135·47	0	93·01	0	101·27	0
	110%	51·97	-0·79	97·44	-2·22	117·26	-1·10	54·87	-0·34	105·18	-2·38	134·13	-1·00	92·83	-0·20	98·86	-2·39
Error rates	This study*	52·39	0	99·66	0	118·55	0	55·05	0	107·75	0	135·47	0	93·01	0	101·27	0
	0%	51·81	-1·10	92·75	-6·92	115·20	-2·83	54·92	-0·24	98·25	-8·82	135·26	-0·16	92·86	-0·16	101·23	-0·05
Equipment lifetime (years)	5	54·32	3·55	106·51	6·44	123·72	4·17	56·85	3·17	123·27	12·59	141·26	4·09	94·81	1·89	115·83	12·56
	10	52·39	0·00	99·66	0·00	118·56	0·00	55·05	0·00	107·75	0·00	135·47	0·00	93·01	0·00	101·27	0·00
Cost of MiSeq	-20%	N/A	N/A	N/A	N/A	117·43	-0·95	N/A	N/A	N/A	N/A	N/A	N/A	N/A	N/A	N/A	N/A
	This study†	N/A	N/A	N/A	N/A	118·56	0·00	N/A	N/A	N/A	N/A	N/A	N/A	N/A	N/A	N/A	N/A
	+20%	N/A	N/A	N/A	N/A	119·70	0·95	N/A	N/A	N/A	N/A	N/A	N/A	N/A	N/A	N/A	N/A
Cost of WGS library preparation	-20%	N/A	N/A	N/A	N/A	114·44	-3·48	N/A	N/A	N/A	N/A	N/A	N/A	N/A	N/A	N/A	N/A
	This study†	N/A	N/A	N/A	N/A	118·56	0·00	N/A	N/A	N/A	N/A	N/A	N/A	N/A	N/A	N/A	N/A
	+20%	N/A	N/A	N/A	N/A	122·69	3·48	N/A	N/A	N/A	N/A	N/A	N/A	N/A	N/A	N/A	N/A
Cost of sequencing cartridge	-20%	N/A	N/A	N/A	N/A	109·24	-7·86	N/A	N/A	N/A	N/A	N/A	N/A	N/A	N/A	N/A	N/A
	This study†	N/A	N/A	N/A	N/A	118·56	0·00	N/A	N/A	N/A	N/A	N/A	N/A	N/A	N/A	N/A	N/A
	+20%	N/A	N/A	N/A	N/A	127·88	7·86	N/A	N/A	N/A	N/A	N/A	N/A	N/A	N/A	N/A	N/A
Sequencing batch size	15	N/A	N/A	N/A	N/A	118·56	0·00	N/A	N/A	N/A	N/A	N/A	N/A	N/A	N/A	N/A	N/A
	17	N/A	N/A	N/A	N/A	113·08	-4·62	N/A	N/A	N/A	N/A	N/A	N/A	N/A	N/A	N/A	N/A
	19	N/A	N/A	N/A	N/A	108·75	-8·27	N/A	N/A	N/A	N/A	N/A	N/A	N/A	N/A	N/A	N/A
Staff grade	Grade 6	N/A	N/A	N/A	N/A	116·70	-1·57	N/A	N/A	N/A	N/A	N/A	N/A	N/A	N/A	N/A	N/A
	Grade 7	N/A	N/A	N/A	N/A	118·56	0·00	N/A	N/A	N/A	N/A	N/A	N/A	N/A	N/A	N/A	N/A

**Total lower**[‡]	N/A	47·04	-10·22	82·25	-17·47	104·02	-12·26	50·15	-8·91	86·71	-19·53	122·61	-9·49	84·93	-8·69	90·36	-10·77
**Total upper**[‡]	N/A	57·26	9·3	110·66	11·04	130·01	9·67	59·86	8·74	119·86	11·24	148·41	9·55	100·99	8·58	112·66	11·25

*Error rates reported during this study were - 1% microscopy, 2% MGIT culture, 10% Cepheid Xpert MTB/RIF, 0·05% species identification (Hain ID), 13% DNA extraction for WGS, 4% WGS, 1·4% WGS data analysis, 10% MIRU-VNTR, DST 0·05%.

†Cost of MiSeq £83,282·00; Cost of Nextera XT library preparation kit £1,649·06; Cost of 300bp v2 MiSeq sequencing cartridge £560·11.

‡Total values are the summation of percentage cost change for the cheapest (lower) and most expensive (upper) of the costs generated during the sensitivity analysis.

References

1. Walker TM, Ip CLC, Harrell RH, et al. Whole-genome sequencing to delineate *Mycobacterium tuberculosis* outbreaks: a retrospective observational study. Lancet Infect Dis 2013;13:137-46.
2. Walker TM, Lalor MK, Broda A, et al. Assessment of Mycobacterium tuberculosis transmission in Oxfordshire, UK, 2007–12, with whole pathogen genome sequences: an observational study. The Lancet Respiratory Medicine 2014;2:285-92.
3. Walker TM, Kohl TA, Omar SV, et al. Whole genome sequencing predicts M. tuberculosis drug susceptibility and resistance: retrospective cohort study. *The Lancet Infectious Diseases* 2015; http://dx.doi.org/10.1016/S1473-3099(15)00062-6.
4. Gardy JL, Johnston JC, Ho Sui SJ, et al. Whole-genome sequencing and social-network analysis of a tuberculosis outbreak. New Engl J Med 2011;364:730-9.
5. Royston PaS, W. Multivariable Model - Building: A Pragmatic Approach to Regression Anaylsis based on Fractional Polynomials for Modelling Continuous Variables. Chichester, UK: Wiley; 2008.
6. Feuerriegel S, Schleusener V, Beckert P, et al. PhyResSE: a Web Tool Delineating *Mycobacterium tuberculosis* Antibiotic Resistance and Lineage from Whole-Genome Sequencing Data. J Clin Microbiol 2015;53:1908-14.
7. Haas WH, Schilke K, Brand J, et al. Molecular analysis of katG gene mutations in strains of *Mycobacterium tuberculosis* complex from Africa. Antimicrob Agents Chemother 1997;41:1601-3.
8. Kolibab K, Derrick SC, Morris SL. Sensitivity to Isoniazid of *Mycobacterium bovis* BCG Strains and BCG Disseminated Disease Isolates. J Clin Microbiol 2011;49:2380-1.
9. Merker M, Kohl TA, Roetzer A, et al. Whole genome sequencing reveals complex evolution patterns of multidrug-resistant *Mycobacterium tuberculosis* Beijing strains in patients. PloS One 2013;8:e82551.
10. Ramaswamy SV, Amin AG, Goksel S, et al. Molecular genetic analysis of nucleotide polymorphisms associated with ethambutol resistance in human isolates of *Mycobacterium tuberculosis*. Antimicrob Agents Chemother 2000;44:326-36.
11. Starks AM, Gumusboga A, Plikaytis BB, Shinnick TM, Posey JE. Mutations at embB codon 306 are an important molecular indicator of ethambutol resistance in *Mycobacterium tuberculosis*. Antimicro Agents Chemother 2009;53:1061-6.
12. Escalante P, Ramaswamy S, Sanabria H, et al. Genotypic characterization of drug-resistant *Mycobacterium tuberculosis* isolates from Peru. Tubercle Lung Dis 1998;79:111-8.
13. Hirano K, Takahashi M, Kazumi Y, Fukasawa Y, Abe C. Mutation in pncA is a major mechanism of pyrazinamide resistance in *Mycobacterium tuberculosis*. Tubercle Lung Dis 1997;78:117-22.
14. Lee KW, Lee JM, Jung KS. Characterization of pncA mutations of pyrazinamide-resistant *Mycobacterium tuberculosis* in Korea. J Korean Med Sci 2001;16:537-43.
15. Wang Q, Lau SKP, Liu F, et al. Molecular epidemiology and clinical characteristics of drug-resistant *Mycobacterium tuberculosis* in a tuberculosis referral hospital in China. PloS One 2014;9:e110209.
16. Liu F, Hu Y, Wang Q, et al. Comparative genomic analysis of *Mycobacterium tuberculosis* clinical isolates. BMC Genomics 2014;15:469.
17. Lau RWT, Ho P-L, Kao RYT, et al. Molecular characterization of fluoroquinolone resistance in *Mycobacterium tuberculosis*: Functional analysis of gyrA mutation at position 74. Antimicrob Agents Chemother 2011;55:608-14.

18. NHS terms and conditions of service handbook. United Kingdom: The NHS Staff Council, 2015. (Accessed 15th January, 2015, at http://www.nhsemployers.org/~/media/Employers/Documents/Pay%20and%20reward/AfC_tc_of_service_handbook_fb.pdf)
